# Tungsten Oxide-Based Z-Scheme for Visible Light-Driven Hydrogen Production from Water Splitting

**DOI:** 10.1021/acscatal.3c01312

**Published:** 2023-06-26

**Authors:** Madasamy Thangamuthu, Kiran Vankayala, Lunqiao Xiong, Stuart Conroy, Xiaolei Zhang, Junwang Tang

**Affiliations:** † Department of Chemical Engineering, University College London, Torrington Place, London WC1E 7JE, U.K.; ‡ Department of Chemical and Process Engineering, University of Strathclyde, Glasgow G1 1XL, U.K.

**Keywords:** green hydrogen, tungsten oxide, Z-scheme, visible photocatalysis, water splitting, DFT calculation

## Abstract

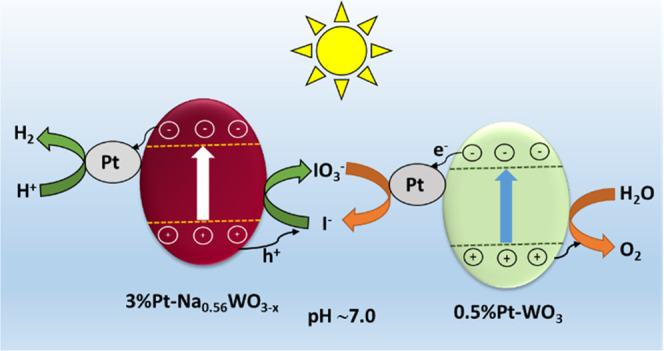

The stoichiometric water splitting using a solar-driven Z-scheme approach is an emerging field of interest to address the increasing renewable energy demand and environmental concerns. So far, the reported Z-scheme must comprise two populations of photocatalysts. In the present work, only tungsten oxides are used to construct a robust Z-scheme system for complete visible-driven water splitting in both neutral and alkaline solutions, where sodium tungsten oxide bronze (Na_0.56_WO_3–*x*_) is used as a H_2_ evolution photocatalyst and two-dimensional (2D) tungsten trioxide (WO_3_) nanosheets as an O_2_ evolution photocatalyst. This system efficiently produces H_2_ (14 μmol h^–1^) and O_2_ (6.9 μmol h^–1^) at an ideal molar ratio of 2:1 in an aqueous solution driven by light, resulting in a remarkably high apparent quantum yield of 6.06% at 420 nm under neutral conditions. This exceptional selective H_2_ and O_2_ production is due to the preferential adsorption of iodide (I^–^) on Na_0.56_WO_3–*x*_ and iodate (IO_3_^–^) on WO_3_, which is evidenced by both experiments and density functional theory calculation. The present liquid Z-scheme in the presence of efficient shuttle molecules promises a separated H_2_ and O_2_ evolution by applying a dual-bed particle suspension system, thus a safe photochemical process.

## 1 Introduction

Hydrogen (H_2_) production from earth-abundant water using sustainable solar energy is imperative to solve global energy demand and environmental issues.^1^ Besides, solar H_2_ is an alternative to gray H_2_ derived from fossil fuels to be used in many industrial processes as feedstock including ammonia synthesis.^2,3^ Solar-driven overall water splitting using semiconductor materials is one of the promising approaches to achieve sustainable production of H_2_ in an economically viable manner.^4^ However, the simultaneous production of H_2_ and O_2_ as an ideal process is extremely challenging in photocatalytic water splitting in the absence of an electric bias. A single photocatalyst with appropriate cocatalysts for pure water splitting has thus met with very limited success due to stringent bandgap requirements.^5,6^ In parallel, there are many studies on the half-reaction of either proton reduction or water oxidation in the presence of an efficient but costly chemical scavenger. Typically, BiVO_4_^7^ or WO_3_^8^ for water oxidation and C_3_N_4_^9^ or Rh dope SrTiO_3_^10^ for proton reduction were reported. A Z-scheme, also known as a dual photoexcitation system, akin to natural photosynthesis comprising H_2_ evolution photocatalyst (HEP) and O_2_ evolution photocatalyst (OEP), should be more efficient and economical for overall water splitting than the single photocatalyst with complicated cocatalysts.^11,12^ It offers an extended choice of semiconductor materials with a narrow bandgap for both half-reactions, enabling it to achieve high solar-to-hydrogen conversion efficiency (STH). Furthermore, it has been predicted that a maximum of 12% STH can be achieved using a single absorber whereas it can be upraised significantly to 22% for Z-scheme-based systems due to much better visible light harvesting.^13^ Typically, HEPs with the more negative conduction band (CB) potential concerning proton reduction (0.0 V vs NHE) and OEPs with the more positive valence band (VB) potential concerning water oxidation (1.23 V vs NHE) are suitable for H_2_ and O_2_ evolutions, respectively. The semiconductor particulate suspension-based Z-scheme system utilizing soluble redox mediator has been highly focused as it is much more simple and cost-effective than the solid Z-scheme,^14^ and more importantly promises to produce H_2_ and O_2_ separately in a dual-bed particle suspension system on a large scale, guaranteeing a safer chemical process compared with others, e.g., a solid Z-scheme.^13,15−17^

Several reports have successfully demonstrated the concept of dual semiconductor photocatalysts suspended in an aqueous solution containing redox couples such as Fe^3+^/^2+^, IO_3_^–^ / I^–^, I_3_^–^/I^–^,^18,19^ etc., as we have summarized in our recent review for light-assisted water splitting.^11^ For example, Abe et al. accomplished an effective overall water splitting using Pt-TiO_2_ (anatase) and Pt-TiO_2_ (rutile) for H_2_ and O_2_ evolutions, respectively, in the presence of IO_3_^–^/I^–^ redox mediator, while under ultraviolet (UV) light.^20^ Subsequently, several narrow-bandgap semiconductors viz. cation-doped SrTiO_3_, graphitic carbon nitride (g-C_3_N_4_), Sm_2_Ti_2_S_2_O_5_, etc., as HEPs, and BiVO_4_, WO_3_, H_2_WO_4_, AgNbO_3_, TaON, etc., as OEPs have been explored and implemented in a Z-scheme system to split water into stoichiometric amounts of H_2_ and O_2_.^4,21,22^ Notably, surface-modified oxynitrides were described to improve the water splitting significantly by absorbing visible light effectively and suppressing the charge carrier recombination; however, oxynitrides are self-photocorrosive, leading to poor photostability.^23^ In another report, a heterojunction based on oxynitrides (Pt-loaded MgTa_2_O_6–*x*_ N_*y*_/TaON) as a HEP was shown to suppress charge carrier recombination, resulting in a drastic enhancement in overall water splitting with a benchmark apparent quantum yield (AQY) of 3.4% at 420 nm when considering a 2-electron process for H_2_ production, in combination with PtO*_x_*/WO*_x_* as an OEP in the presence of IO_3_^–^/I^–^ as redox mediator.^24^ Similarly, the AQY of a Z-scheme photocatalytic system at 420 nm was often measured.^10,25−29^ Though substantial advances have been made so far, a significant breakthrough is yet to come. Hence, intensive research is underway by developing new materials^30^ with special surface properties, high crystallinity, fewer defects,^31^ spatial separation of reduction and oxidation sites, cocatalyst loading,^32^ controlled morphology, heterojunction formation, etc., to achieve a high AQY.

Herein, we have developed a new tungsten oxide-only Z-scheme (WOZ) system for overall water splitting, in which both the HEP and OEP are tungsten oxides, i.e., sodium tungsten oxide bronze (Na_0.56_WO _3–*x*_) as the HEP and two-dimensional (2D) WO_3_ nanosheets as the OEP in the presence of IO_3_^–^/I^–^ redox couple. Tungsten oxide-based materials have been widely studied but only as photoanodes or water oxidation photocatalysts due to their conduction band being too positive to meet proton reduction.^8,33,34^ Moreover, WO_3_ absorbs ∼12% of the solar spectrum and possesses a moderate hole-diffusion length (∼150 nm) in addition to impressive electron mobility (∼12 cm^2^ V^–1^ s^–1^).^34^ Numerous stable nonstoichiometric WO_3_ materials could be realized by altering the lattice oxygen vacancies to attain interesting optoelectronic/catalytic properties. To the best of our knowledge, a Z-scheme system composed of one type of oxide photocatalysts for visible-driven-pure water splitting has not been reported. Very recently upraising the CB position of WO*_x_* to a more negative potential was shown, e.g., by creating oxygen vacancies^35^ or surface defects engineering.^36^ Here, we prepared a new and stable WO*_x_*-based HEP, Na_0.56_WO_3–*x*_. Interestingly, it can be coupled with WO_3_ nanosheets as an OEP to form a robust Z-scheme, which demonstrates efficient pure water splitting under light irradiation. Compared with the bulk materials, WO_3_ nanosheets provide more exposed active sites due to their sheet-like morphology. Such a novel Z-scheme generates H_2_ and O_2_ stoichiometrically, demonstrating a high AQY of 6.06% at 420 nm, under light irradiation and ambient conditions.

## 2 Results and Discussion

### 2.1 Characterization of the Photocatalysts

#### 2.1.1 Hydrogen Evolution Photocatalyst (HEP)

**Figure 1:**
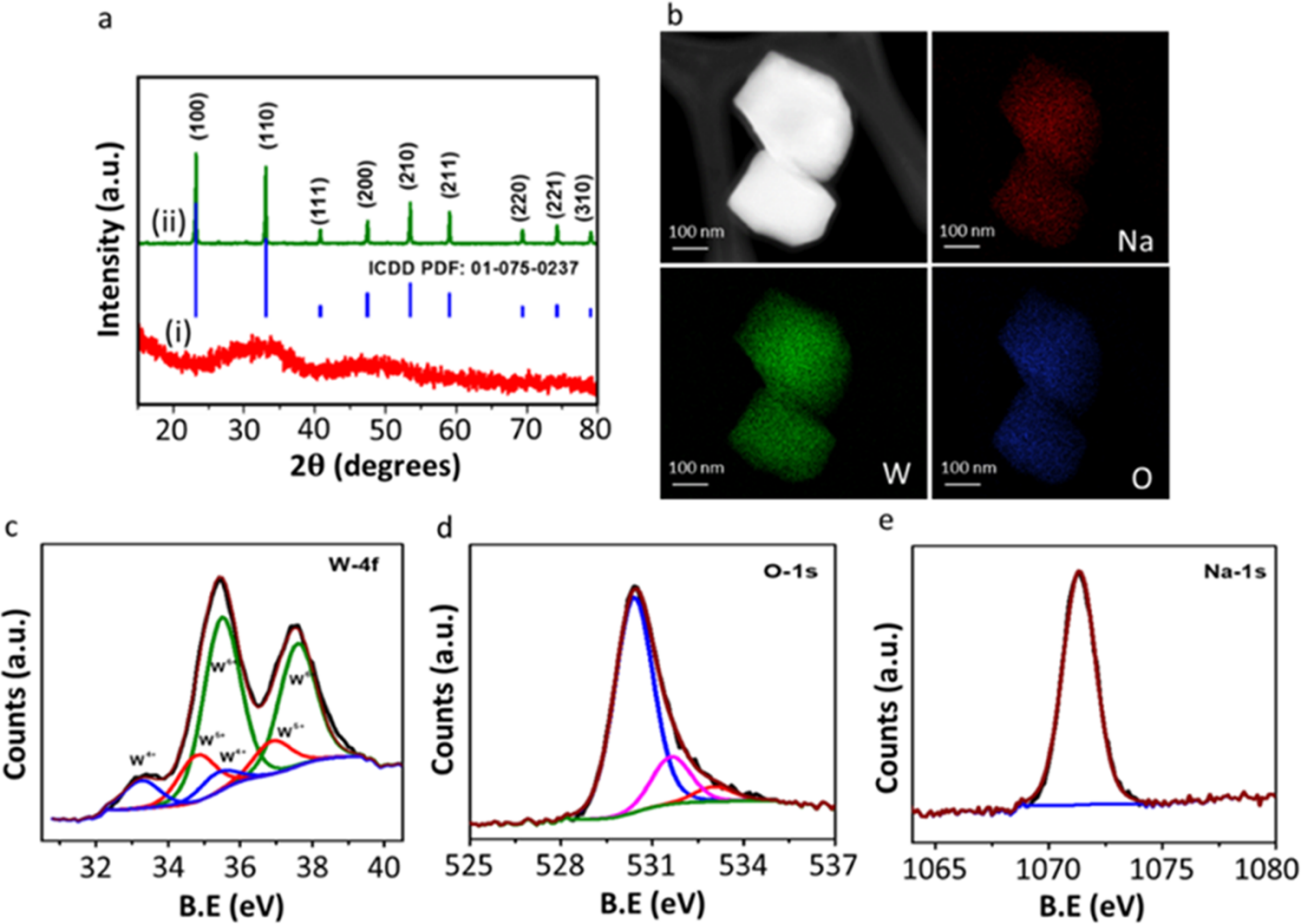
Characterization of HEP. (a) Powder XRD patterns of the (i) as-synthesized Na*_x_*WO_3–*x*_ (NWO), a standard pattern of the cubic Na*_x_*WO_3–*x*_ with PDF file no. 01-75-0237 (blue) and (ii) annealed Na*_x_*WO_3–*x*_ (Na_0.56_WO_3–*x*_); (b) HAADF TEM image of Na_0.56_WO_3–*x*_ and the corresponding elemental mapping of Na (red), W (green), and O (blue); and (c–e) XPS spectra of W-4f, O-1s, and Na-1s regions in Na_0.56_WO_3–*x*_, respectively.

Na*_x_*WO_3–*x*_ was prepared under ambient conditions by treating sodium tungstate with sodium borohydride (see details in the Experimental Section). The obtained powders were characterized using various physicochemical techniques. Figure 1a shows the powder XRD patterns of the as-synthesized samples and samples annealed at 800 °C under a N_2_ atmosphere. It is clearly seen that the as-synthesized sample is amorphous as it has very broad diffraction peaks. However, the annealed sample exhibits sharp strong peaks, indicating that annealing imparted crystallinity to the sample, as expected. These peaks can be assigned to the cubic Na*_x_*WO_3–*x*_ (*Pm-*3*m*) (Figure S1), matching well with the standard pattern (PDF No. 01-75-0237). The Na content present in the annealed Na*_x_*WO_3–*x*_ was determined to be around 0.56 using a microwave plasma-atomic emission spectrometer, which is also verified using the experimentally (XRD) measured lattice parameter (*a*_o_) according to the eq 1,^37,38^ so this sample is denoted Na_0.56_WO_3–*x*_.
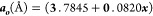
1

Figure S2 represents transmission electron microscopy (TEM) images of the as-synthesized HEP, indicating that the sample is composed of interconnected spheres with dimensions around 100–150 nm. The high-resolution TEM (HRTEM) image of the as-synthesized sample shows the absence of crystallinity, demonstrating the amorphous nature. After annealing, the amorphous sample becomes crystalline, which is obvious from the TEM image (Figure S3), and the Pt nanoparticles distribution on the surface of the Na_0.56_WO_3–*x*_ reveals the successful cocatalyst loading. Furthermore, the new crystalline Na_0.56_WO_3–*x*_ lattice was identified as (100), and the photodeposited Pt nanoparticles show (200) lattice (Figure S4). The elemental mapping of Na_0.56_WO_3–*x*_ shows the uniform distribution of Na, W, and O elements (Figure 1b). The surface oxidation states of Na_0.56_WO_3–*x*_ were assessed using X-ray photoelectron spectroscopy (XPS). The survey spectrum is shown in Figure S5. Figure 1c–e represents the deconvoluted core-level XPS spectra of W-4f, O-1s, and Na-1s recorded for Na_0.56_WO_3–*x*_. As shown in Figure 1c, the presence of three doublets (4f_7/2_ and 4f_5/2_) in the W-4f spectra of Na_0.56_WO_3–*x*_ unveils the presence of tungsten in multiple oxidation states. For instance, it shows peaks at binding energy (BE) values around 33.3 and 35.4 eV, 34.8 and 36.9 eV, and 35.5 and 37.6 eV. The first set of doublets corresponds to the presence of W^4+^, while the second and third doublets disclose the presence of W^5+^ and W^6+^ species, respectively. The deconvoluted O-1s XPS consists of three peaks at BE values 530.4, 531.6, and 533.1 eV^37^ (Figure 1d). The most intense peak at 530.4 eV is attributed to lattice oxygen (O^2–^) surrounded by W atoms in the Na_0.56_WO_3–*x*_ lattice. The origin of the peak at 531.6 eV is due to oxygen (OH^–^) (the adsorbed OH^–^) in the regions of oxygen vacancies (due to W^5+^/W^4+^ species) within the Na_0.56_WO_3–*x*_ matrix. In these oxygen-deficient sites, OH groups are known to bond with metal cations to compensate for the charge. The peak located at 533.1 eV could be due to the presence of adsorbed H_2_O on the surface of Na_0.56_WO_3–*x*_.^37^ The deconvoluted Na-1s XPS shows a single peak at 1071.2 eV (Figure 1e), in agreement with an earlier study.^39^ A similar set of peaks are observed after cocatalyst Pt loading, Pt- Na_0.56_WO_3–*x*_ (see Table S1).

#### 2.1.2 Oxygen Evolution Photocatalyst (OEP)

**Figure 2:**
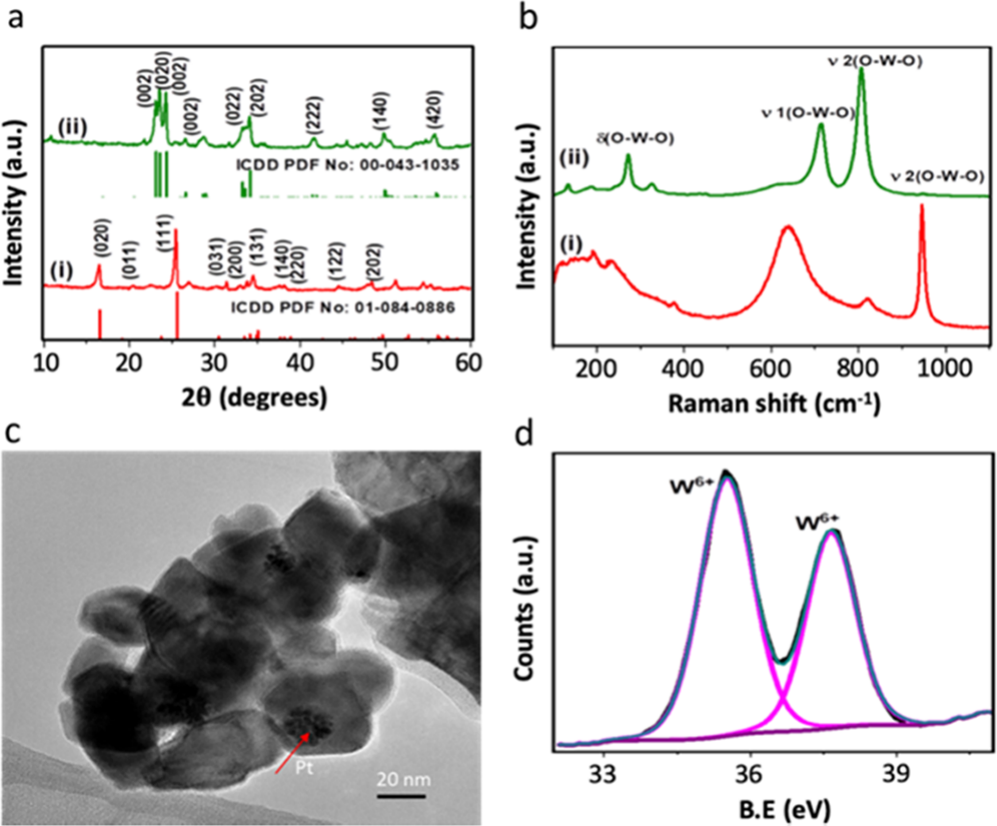
Characterization of OEP. (a) XRD patterns of (i) as-synthesized WO_3_·H_2_O and (ii) annealed (WO_3_ nanosheets) samples and their corresponding standard ICDD patterns; (b) Raman spectra of (i) WO_3_·H_2_O and (ii) WO_3_ nanosheets recorded using a 532 nm green laser; (c) TEM image of Pt-WO_3_ and the presence of Pt nanoparticles on the surface of WO_3_ is indicated with an arrow; and (d) XPS spectra corresponding to the W-4f region of WO_3_ nanosheets.

2D WO_3_ nanosheets as an OEP in the Z-scheme system were obtained by thermal annealing of 2D tungstic acid (WO_3_·H_2_O) under an air atmosphere at 500 °C (see the Experimental Section for details). The conversion of tungstic acid into tungsten trioxide was studied using XRD and Raman spectroscopy. The powder XRD pattern of WO_3_ nanosheets along with its precursor WO_3_·H_2_O are shown in Figure 2a. The as-synthesized WO_3_·H_2_O nanosheets are crystalline and the XRD pattern matches well with the standard pattern (PDF No: 01-084-0886) for orthorhombic (*Pmnb*) hydrated WO_3_ with strong diffraction peaks for (111), (020), and (131) planes, in agreement with the earlier report.^40^

After annealing it in the air at 500 °C for 3 h, the formation of monoclinic (**P*21/*n**) WO_3_ nanosheets is observed, corresponding to the standard pattern (PDF No: 00-043-1035). It confirms the transformation of orthorhombic WO_3_·H_2_O into monoclinic WO_3_ due to annealing (Figure S6). This phenomenon is further verified by Raman spectroscopy, as shown in Figure 2b. The Raman spectra of WO_3_·H_2_O show three peaks at ∼642, ∼820, and ∼946 cm^–1^, corresponding to the first W–O–W, [ν1(W–O–W)], second W–O–W [ν2(W–O–W)], and terminal W=O stretching vibrations, respectively. The sharp peaks at ∼642 and ∼946 cm^–1^ confirm that the as-synthesized nanosheets are hydrated WO_3_ (WO_3_·H_2_O). The Raman spectra of WO_3_ nanosheets (Figure 2b) show two major peaks at ∼714 and ∼808 cm^–1^, corresponding to the W–O–W stretching modes, confirming the formation of monoclinic WO_3_. The other notable difference in WO_3_ nanosheet spectra is the absence of terminal W=O stretching mode at 946 cm^–1^ compared to WO_3_·H_2_O. Besides, a strong peak appears at 272 cm^–1^ along with two low intense peaks at around 192 and 325 cm^–1^, which are corresponding to W–O–W bending modes of WO_3_.^41^ The ratio of *I*_ν2(W-O-W)_/*I*_ν(W_=_O)_ can provide information about *x* in WO_3_·*x*H_2_O as suggested earlier.^40^ As expected the ratio is lower for WO_3_·H_2_O, while it is higher for WO_3_ nanosheets, validating the dehydration of WO_3_·H_2_O. The layered morphology of WO_3_·H_2_O nanosheets is evident from the TEM images, as shown in Figure S7. The EDX spectra validate that the material is mainly made of W and O elements. Figure 2c shows the TEM image of Pt-WO_3_, which shows the presence of Pt nanoparticles on the surface of the WO_3_ mixture composed of nanosheets and nanoparticles (denoted WO_3_ nanosheets afterward). The deconvoluted W-4f XPS of WO_3_ shown in Figure 2d displays a doublet at BE values 37.5 and 37.6 eV suggesting that the W is present at +6 oxidation state only. These results suggest that W has a single oxidation state in WO_3_. The presence of W^+^6 also confirms the formation of WO_3_ upon annealing of WO_3_·H_2_O, complementing XRD, and Raman measurements.

#### 2.1.3 Band Structure of HEP and OEP

**Figure 3:**
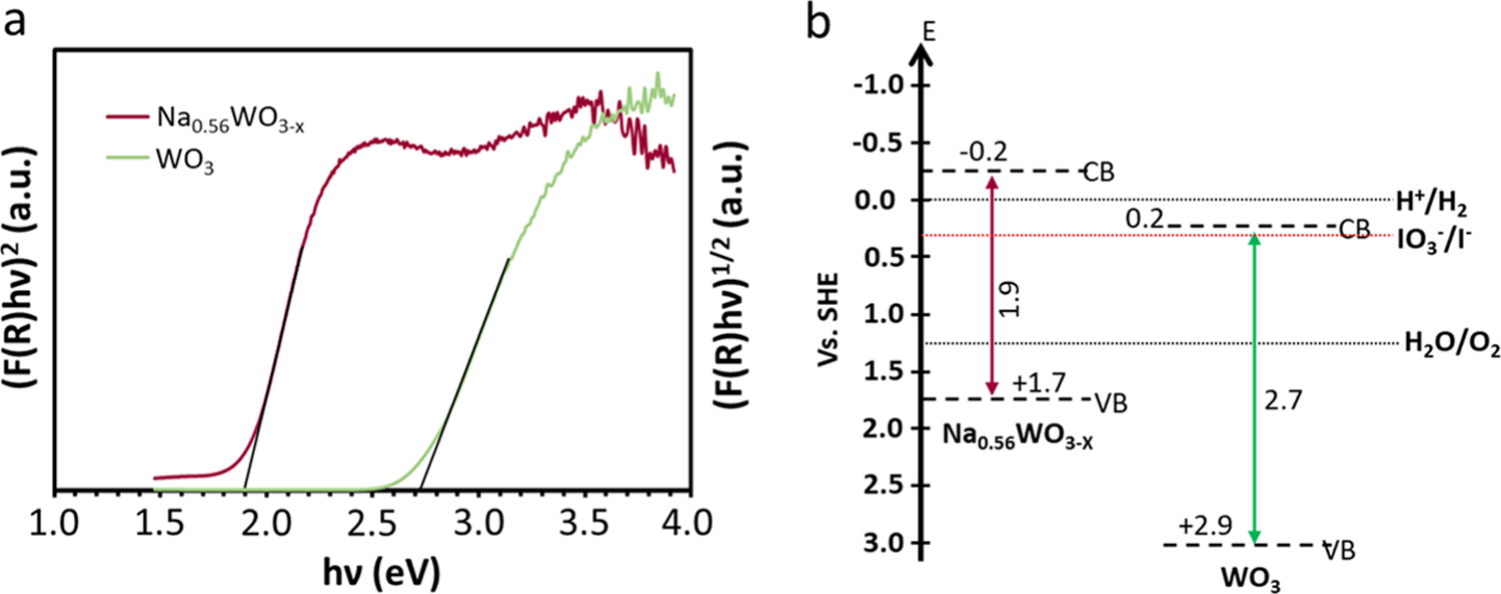
(a) Tauc plots of WO_3_ nanosheets, an indirect bandgap semiconductor, and Na_0.56_WO_3–*x*_, a direct bandgap semiconductor; (b) the proposed band diagram for Na_0.56_WO_3–*x*_- and WO_3_-based Z-scheme photocatalytic water splitting system vs SHE.

UV–Vis diffuse reflectance spectroscopy (DRS) was used to disclose the bandgap of the investigated photocatalysts. The optical absorbance spectra of Na_0.56_WO_3–*x*_ and WO_3_ nanosheets are shown in Figure S8. It is clearly seen that both materials absorb visible light efficiently and the Tauc plots of Na_0.56_WO_3–*x*_ and WO_3_ (Figure 3a) reveal a bandgap of 1.9 and 2.7 eV, respectively. To explicitly determine the band positions, we have measured the CB by photoelectrochemistry and Mott–Schottky measurement. The photocurrent onset potential measurement results are shown in Figure S9. The measured potential against Ag/AgCl was converted to SHE using *E*_(SHE)_ = *E*_Ag/AgCl_ + *E*_Ag/AgCl_^0^. The WO_3_ has a CB potential of +0.2 V, which is consistent with the reported^42^ and the Na_0.56_WO_3–*x*_ has CB at −0.2 V. To validate it further, we have tested the CB potential of the Na_0.56_WO_3–*x*_ and WO_3_ using the Mott–Schottky method (Figure S10), and the observed results agree with the photocurrent onset potential measurement. In addition, we have performed a control experiment to test the water oxidation property of Na_0.56_WO_3–*x*_ in the presence of IO_3_^–^ and observed a tiny amount of O_2_ evolved (0.3 μmol h^–1^), 60 times less active compared to WO_3_ (17.9 μmol h^–1^). Such a small O_2_ evolution rate of Na_0.56_WO_3–*x*_ reveals the less positive VB potential of Na_0.56_WO_3–*x*_ compared to WO_3_. Thus, it is essential to engineer a Z-scheme composed of Na_0.56_WO_3–*x*_ and WO_3_ for complete water splitting. Accordingly, the energy band diagram was drawn, as shown in Figure 3b. The CB of WO_3_ is too positive to reduce protons into H_2_, which is essential to form an efficient Z-scheme system as it is close to the VB of the HEP when coupling Na_0.56_WO_3–*x*_ with WO_3_. The deep VBM of WO_3_ nanosheets and negative CBM of Na_0.56_WO_3–*x*_ suggest that it is highly suitable for water oxidation, and reduction reactions, respectively. More importantly, these well-matched band positions promise a new Z-scheme composed of tungsten oxides.

### 2.2 Photocatalytic H_2_ Evolution

**Figure 4:**
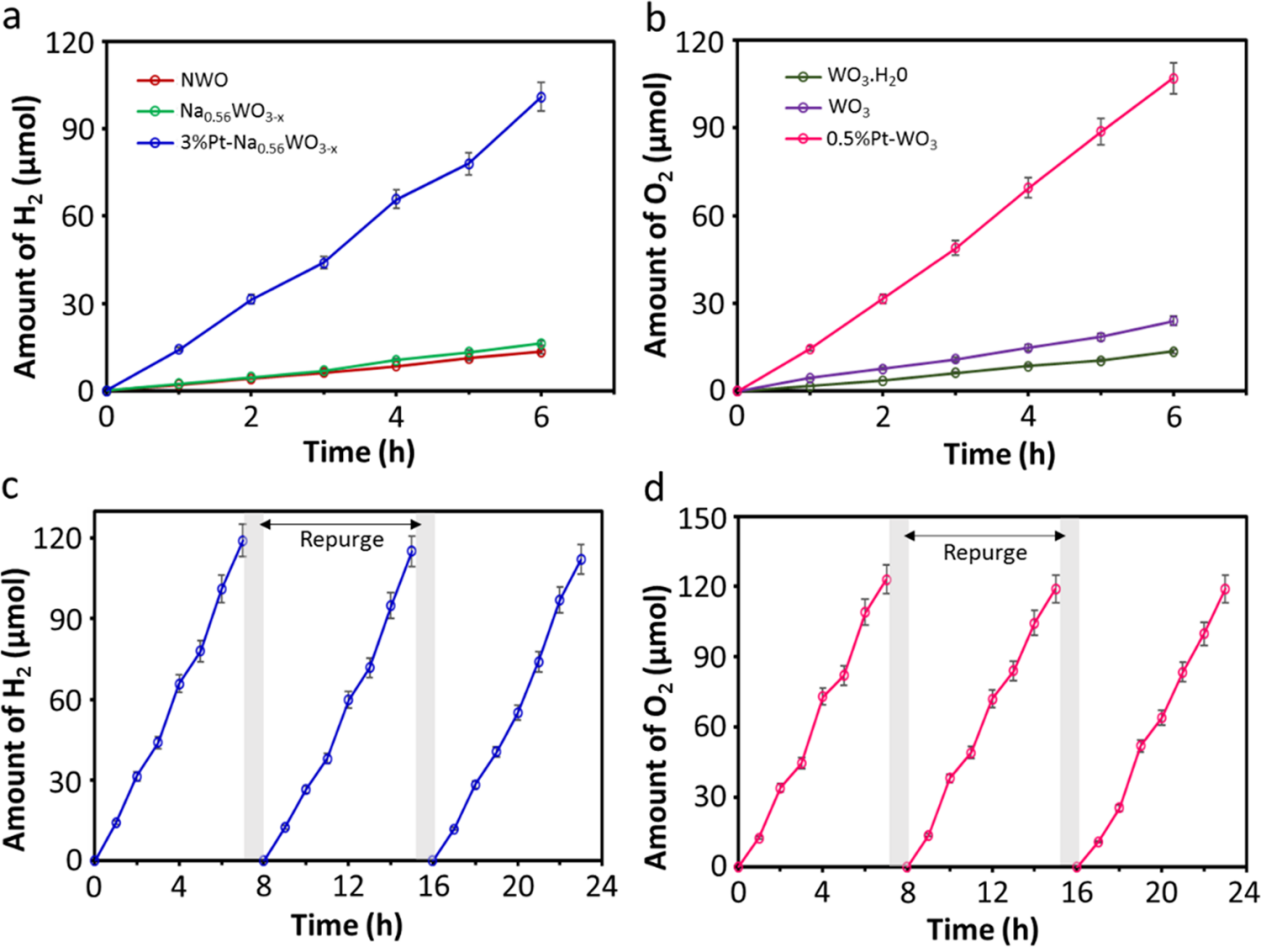
Photocatalytic H_2_ and O_2_ evolution half-reactions. (a) H_2_ evolution from water (pH 7.0) using 10 mg of NWO-based materials (NWO, Na_0.56_WO_3–*x*_, and 3wt%Pt-Na_0.56_WO_3–*x*_) containing 5 mM NaI as a hole scavenger under a full-arc condition for 6 h; (b) O_2_ evolution from water (pH 8.5) using 10 mg of WO_3_-based materials (WO_3_·H_2_O, WO_3_ nanosheets, and 0.5%Pt-WO_3_ nanosheets) containing 5 mM NaIO_3_ as an electron scavenger under a full-arc condition for 6 h; (c) the H_2_ evolution stability study on 3%Pt-Na_0.56_WO_3–*x*_ in an aqueous solution (pH 7.0) containing 5 mM NaI under a full-arc condition for three consecutive 7 h runs; and (d) the O_2_ evolution stability study on 0.5%Pt-WO_3_ nanosheets in an aqueous solution (pH 8.5) containing 5 mM NaIO_3_ under a full-arc condition for three 7 h runs; Ar gas was purged after each run.

To verify the photocatalytic ability of Na_0.56_WO_3–*x*_-based materials for H_2_ production, experiments were performed in aqueous solutions containing NaI (pH ∼7.0) as a hole scavenger under Xe lamp irradiation, and the results are shown in Figure 4a. The compelling observation from Figure 4a is that the NWO and Na_0.56_WO_3–*x*_ can produce H_2_ without any cocatalysts, which is otherwise difficult with WO_3_-based materials due to the inappropriate CB position concerning the H_2_ evolution potential, suggesting that both NWO and Na_0.56_WO_3–*x*_ can be used as a photocatalyst for H_2_ production from water. Both NWO (amorphous) and Na_0.56_WO_3–*x*_ (crystalline) photocatalysts produce H_2_ at a rate of 2.2 and 2.5 μmol h^–1^, respectively. Subsequently, the effect of pH on H_2_ evolution was studied to optimize the condition at which NWO-based materials yield higher rates of H_2_ production. As shown in Figure S11, the Na_0.56_WO_3–*x*_ shows better activity than NWO from neutral to weakly alkaline solution, where the H_2_ evolution rate was obtained from the first 6 h run. Furthermore, we have tested the chemical stability of Na_0.58_WO_3–*x*_ for H_2_ evolution at pH 8.5 and 10.5 by running four 6 h reaction cycles (Figure S12). The initial rate of H_2_ evolution does not change significantly at both pH values, but from the third cycle onward, the H_2_ evolution rate starts to reduce and saturates in the fourth cycle only at pH 10.5. This suggests that the present HEP is highly stable under a weakly alkaline condition but not stable at a strong alkaline condition for a prolonged run. Hence, well-crystallized Na_0.56_WO_3–*x*_ was selected for further studies. To improve the activity, 3wt%-Pt (cocatalyst) particles were loaded on the surface of Na_0.58_WO_3–*x*_ by photodeposition (see Experimental Section for details), which significantly improves the H_2_ evolution to a rate of 16.8 μmol h^–1^ at pH 7.0. It is worth noting that after loading the cocatalyst (3%Pt-Na_0.56_WO_3–*x*_), the best activity was observed at pH 7.0. This is anticipated as the cocatalysts extract the electron efficiently and minimize the recombination with holes. The H_2_ evolution reaction in the presence of I^–^ ions at pH 7.0 can be represented by the following equations.
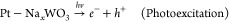
2
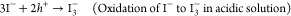
3
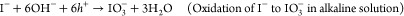
4
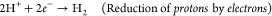
5

The stability of the 3%Pt-Na_0.56_WO_3–*x*_ for prolonged H_2_ evolution was investigated by measuring the H_2_ evolution for 21 h (composed of three consecutive 7 h runs) and no significant change in the H_2_ evolution rate is observed, as shown in Figure 4c, suggesting the robustness of Na_0.56_WO_3–*x*_ under neutral pH. We further validated the stability of the HEP by testing the compositional changes using XPS after three consecutive photocatalytic runs, as shown in Figure S13. No obvious changes were observed in the deconvoluted spectra of O-1S and Na-1S; however, a small reduction in the W^4+^ signal was observed, while the signals for W^5+^ and W^6+^ are stable. The identical H_2_ evolution suggests that W^5+^ and W^6+^ are the species dominating water reduction under the present experimental conditions. Furthermore, the XRD spectra of the 3%Pt-Na_0.56_WO_3–*x*_ before and after the photocatalysis show (Figure S14a) no significant changes, which again confirms the robustness of the HEP under neutral pH.

### 2.3 Photocatalytic O_2_ Evolution

Subsequently, we investigated the photocatalytic O_2_ evolution half-reaction using WO_3_ under a full-arc condition in water containing IO_3_^–^ ions as a sacrificial electron acceptor. As expected, a significant amount of O_2_ evolution was observed at ∼pH 8.5 using both WO_3_·H_2_O and WO_3_, but monoclinic WO_3_ nanosheets show better activity than orthorhombic WO_3_·H_2_O (Figure 4b). Thereafter, WO_3_ nanosheets were loaded with 0.5 wt % Pt cocatalyst (Pt-WO_3_), leading to an O_2_ evolution rate of 17.9 μmol h^–1^, which is superior to cocatalyst-free WO_3_ (3.8 μmol h^–1^) and WO_3_·H_2_O (2.2 μmol h^–1^). The present Pt-WO_3_ water oxidation activity is comparable to the best activity reported previously,^43−45^ thanks to the advantages of nanosheet morphology. The O_2_ evolution using Pt-WO_3_ from weakly alkaline water containing IO_3_^–^ ions can be represented by the following equations.
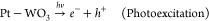
6
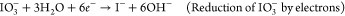
7
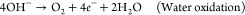
8

In the absence of a photocatalyst or an electron acceptor (IO_3_^–^), no O_2_ evolution is observed. The H_2_ evolution activity of the Pt-WO_3_ was also monitored using I^–^ as the hole scavenger to examine the suitability of WO_3_ for a potentially efficient Z-scheme for water splitting. As anticipated, negligible H_2_ evolution is observed (Figure S15), in agreement with the CB position of WO_3_ measured in Figure 3d, which is not appropriate for water reduction, suggesting that the Pt-WO_3_ is highly selective for the water oxidation half-reaction. The stability of Pt-WO_3_ nanosheets for prolonged O_2_ evolution at pH 8.5 was examined for 21 h (composed of 3 consecutive 7-h runs), and no significant decrease in O_2_ evolution rate is observed, as shown in Figure 4d, suggesting that WO_3_ is a robust photocatalyst under weakly alkaline conditions. In addition, no changes were observed in the XRD of Pt-WO_3_ before and after photocatalysis (Figure S14b), which further ensures stability. Furthermore, we also tested the stability of WO_3_ at pH 10.5 and observed no change in the beginning of the reaction, similar to the HEP, but over a prolonged run, the O_2_ evolution rate was significantly reduced (Figure S16). This strongly suggests that the present OEP is stable only under weak alkaline pH and not stable in strong alkaline solutions.

### 2.4 Z-Scheme Overall Water Splitting

The overall water splitting activity of the new Z-scheme system composed of only tungsten oxides, i.e., 3%Pt-Na_0.56_WO_3–*x*_ as the HEP and 0.5%Pt-WO_3_ as the OEP was then tested under full-arc as well as visible light conditions. When each photocatalyst is tested independently, only H_2_ or O_2_ evolution was observed (Table 1, run 1 and 2). When the system is composed of both the HEP and OEP in an aqueous solution containing I^–^ as a redox mediator, the successful evolution of H_2_ and O_2_ was observed simultaneously (Table 1, run 6). In the case of an aqueous solution containing IO_3_^–^ as a redox mediator, the simultaneous evolution of H_2_ and O_2_ was not observed. Instead, only O_2_ evolution was noted (Table 1, run 3), suggesting that the reduction of IO_3_^–^ to I^–^ is slower, either because I^–^ cannot desorb easily from the OEP or IO_3_^–^ reduction strongly competes with proton reduction to H_2_, which will be discussed later. Both H_2_ and O_2_ production rates are highly dependent on the pH of the solution, which is shown in Table 1 that at pH ∼3.0 acidic condition, poor H_2_ and no O_2_ evolutions were observed in the I^–^ aqueous solution (Table 1, run 4). This could be due to the oxidation of I^–^ to mainly I_3_^–^, which is a poor electron scavenger compared to IO_3_^–^ at pH 3.0, as listed in eq 3 against eq 4, and is preferable in a neutral/alkaline solution and in turn hinders the establishment of the IO_3_^–^/I^–^ redox system.^46^ Similar results were observed while using Fe^2+^ as a redox mediator (Table 1, run 5). At pH ∼7.0, in the presence of I^–^, the evolution of both H_2_ and O_2_ gases was attained (Table 1, run 6); however, the stoichiometric ratio of H_2_ to O_2_ has not been achieved. Further, when the reactions were carried out at pH ∼8.5 (Table 1, run 7), simultaneous productions of H_2_ and O_2_ were observed; however, yet again in nonstoichiometric quantities. Subsequently, we performed optimization studies by varying the weight ratio of HEP to OEP at pH ∼7.0. A weight ratio of 1:1 (HEP:OEP, by weight) has been found to produce H_2_ and O_2_ gases simultaneously with a rate of 14 μmol h^–1^ (2800 μmol h^–1^ g^–1^) and 6.9 μmol h^–1^ (1380 μmol h^–1^ g^–1^), respectively, at a stoichiometric molar ratio of 2:1 (Table 1, run 8).

**Table 1:** Photocatalytic Water Splitting over the WO*_x_* Catalysts (Runs 3 to 7, and 10: 10 mg of HEP and 5 mg of OEP, runs 8, 9, and 11: 5 mg of HEP and 5 mg of OEP) Suspended in an Aqueous Solution Performed under Different Experimental Conditions

run	HEP	OEP	mediator	pH	weight ratio	HER (μmol h^–1^)	OER (μmol h^–1^)
1	3%Pt-Na_0.56_WO_3–*x*_	-	NaI	7.0	-	16.8	-
2	-	0.5%Pt-WO_3_	NaIO_3_	8.5	-	-	17.9
3	3%Pt-Na_0.56_WO_3–*x*_	0.5%Pt-WO_3_	NaIO_3_	7.0	2:1	-	11.3
4	3%Pt-Na_0.56_WO_3–*x*_	0.5%Pt-WO_3_	NaI	3.0	2:1	3.0	-
5	3%Pt-Na_0.56_WO_3–*x*_	0.5%Pt-WO_3_	FeCl_2_	3.0	2:1	2.8	-
6	3%Pt-Na_0.56_WO_3–*x*_	0.5%Pt-WO_3_	NaI	7.0	2:1	14.6	4.8
7	3%Pt-Na_0.56_WO_3–*x*_	0.5%Pt-WO_3_	NaI	8.5	2:1	13.9	5.3
8	3%Pt-Na_0.56_WO_3–*x*_	0.5%Pt-WO_3_	NaI	7.0	1:1	14.0	6.9
9	3%Pt-Na_0.56_WO_3–*x*_	0.5%Pt-WO_3_	NaI	10.5	1:1	10.7	7.9
10	3%Pt-Na_0.56_WO_3–*x*_	0.5%Pt-WO_3_	NaI	10.5	2:1	9.6	4.9
11	3%Pt-Na_0.56_WO_3–*x*_	0.5%Pt-WO_3_	-	7.0	1:1	-	-

**Figure 5:**
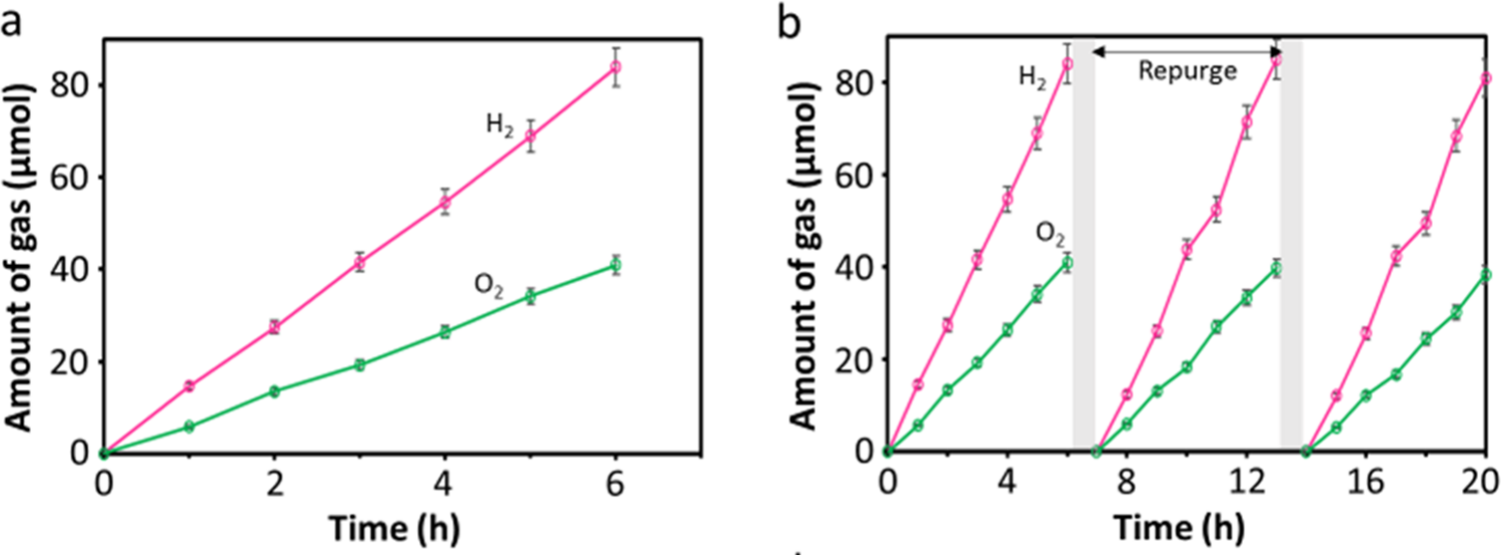
Stoichiometric water splitting with error bar after three measurements. (a) WOZ water splitting system composed of 10 mg of 3wt%Pt-Na_0.56_WO_3–*x*_ as the HEP and 10 mg of 0.5wt%Pt-WO_3_ nanosheets as the OEP under the full-arc condition at pH 7.0 and 5 mM NaI as a redox mediator; (b) the stability study on 3wt%Pt-Na_0.56_WO_3–*x*_–NaI–0.5wt%Pt-WO_3_ under the same photocatalytic conditions for three successive 6 h runs.

The temporal gas evolution is shown in Figure 5a and the consecutive run is represented in Figure 5b, suggesting that the present system is not only efficient but also rather stable under the present experimental conditions. We also tested the present WO*_x_*-based Z-scheme (WOZ) activity at pH 10.5 and observed no stoichiometric gas evolution at a 1:1 mass ratio (Table 1, run 9), whereas at a mass ratio of 2:1, stoichiometric gas evolution was observed (Table 1, run 10), proving that the tungsten oxide-based Z-scheme can split water at both neutral and weakly alkaline conditions. In the absence of a redox mediator (Table 1, run 11), no gas evolution was observed, indicating that the I^–^ is crucial for the Z-scheme. The performance of the present Z-scheme water splitting system under visible-light irradiation (λ ≥ 420 nm) was also measured, as shown in Figure S17. Again, the simultaneous production of H_2_ and O_2_ with a rate of 7.4 and 3.6 μmol h^–1^ is monitored, and the gas amount produced remains almost linear increase with time. The H_2_ production activity of the Na_0.56_WO_3–*x*_ has been tested under half-reaction conditions at higher wavelengths using 600 and 650 nm monochromatic filters. The obvious H_2_ evolution was observed under half-reaction conditions with a rate of 0.9 and 0.27 μmol h^–1^, respectively (Figure S18), but under Z-scheme working conditions, pure water splitting was not observed (generation of both H_2_ and O_2_) as WO_3_ is silent at these very long wavelengths. The AQY for H_2_ production from water using the proposed WOZ system was assessed three times and the average was reported. At 420 nm, an AQY of 6.06% (Figure S19) for water splitting was determined by considering the 2-electron process involved for one molecule of H_2_ evolution (see Experimental Section for details, and in some works of the literature, the 4-electron process was used to calculate the AQY.^47^ Using that method, the AQY is 12% herein). The H_2_ and O_2_ evolution rates under 420 nm monochromatic irradiation are shown in Figure S20. The Z-scheme photocatalytic activity of the present works was compared with the reported representative studies (Table S2).

### 2.5 Adsorption Studies

As the current Z-scheme works very efficiently, we investigated the shuttle molecule′s function in detail. First, both HEP and OEP were tested for H_2_ production in the presence of hole scavenger I^–^ (Figure S15). One can see that the H_2_ is produced on the 3%Pt-Na_0.56_WO_3–*x*_ but not on the 0.5%Pt-WO_3_ nanosheets, proving that the CB of WO_3_ nanosheets is not appropriate for proton reduction while the CB of Na_0.56_WO_3–*x*_ is negative enough to reduce the proton to H_2_ (Figure 3d). The selectivity of the OEP, 0.5%Pt-WO_3_ for the O_2_ evolution half-reaction is then verified, where I^–^ oxidation (I^–^ → IO_3_^–^) might compete with water oxidation by consuming the photogenerated holes from the OEP. We examined this by adding 1 mM I^–^ anions at 3 h during the ongoing water oxidation half-reaction in an aqueous solution (pH ∼8.5) containing 5 mM IO_3_. Interestingly, no influence was observed by the extra I^–^ anions on the water oxidation reaction (Figure S21), strongly indicating that the nanosheets OEP can produce O_2_ highly selectively even in the presence of efficient hole scavenger I^–^. To disclose the reason behind this, we have studied the adsorption behavior of IO_3_^–^ and I^–^ anions onto the OEP by measuring their concentrations before and after the addition of 0.5%Pt-WO_3_ powder to the solution under dark conditions (see Experimental Section for details).

**Figure 6:**
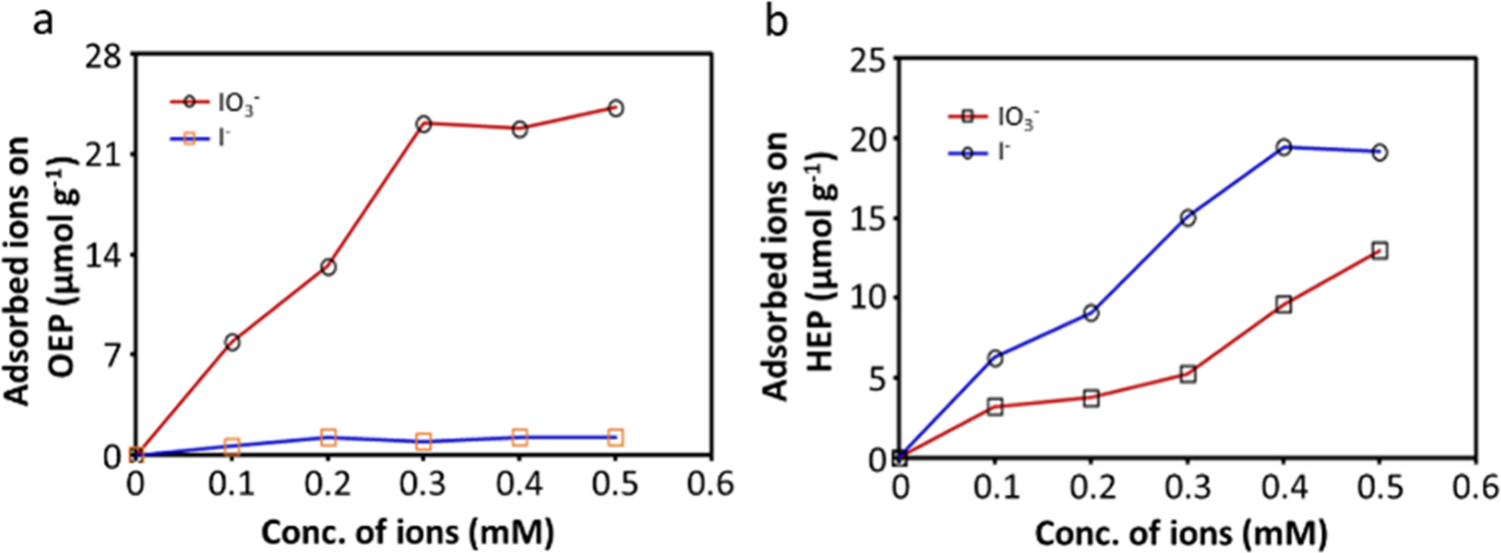
Adsorption study. (a) Adsorption behavior of IO_3_^–^ and I^–^ anions on the surface of 0.5wt%Pt-WO_3_ (OEP) powder suspended in an aqueous solution at pH ∼8.5 under dark conditions; (b) adsorption behavior of IO_3_^–^ and I^–^ anions on 3wt%Pt-Na_0.56_WO_3–*x*_ (HEP) powder suspended in an aqueous solution at pH ∼7.0 under dark conditions.

As shown in Figure 6a, the IO_3_^–^ ions are adsorbed preferentially onto the OEP surface whereas I^–^ shows poor adsorption, confirming that the water oxidation is dominant on the OEP rather than the oxidation of I^–^ to IO_3_^–^. On the other hand, the crucial IO_3_^–^ ions reduction is guaranteed due to their strong adsorption. Therefore, the couple of IO_3_^–^/I^–^ is the ideal charge mediator for a Z-scheme containing the WO_3_ nanosheets. Similarly, we also tested the competitive reaction between water reduction and IO_3_^–^ reduction (IO_3_^–^ → I^–^) driven by the photogenerated electrons on 3%Pt-Na_0.56_WO_3–*x*_, HEP. As shown in Figure S22, we added two distinct concentrations of IO_3_^–^ (100 μM and 1 mM) after 3 h during the ongoing water reduction half-reaction and noticed no significant change upon the addition of 100 μM of IO_3_^–^. However, a substantial deceleration of H_2_ evolution was observed when adding 1 mM IO_3_^–^. These results suggest that at higher IO_3_^–^ concentrations, the photogenerated electrons from the HEP are also consumed by IO_3_^–^ ions, in good agreement with the earlier report^20^ where a similar phenomenon was observed.

To understand further, we observed the competitive adsorption behavior of I^–^ and IO_3_^–^ on 3%Pt- Na_0.56_WO_3–*x*_. As shown in Figure 6b, both I^–^ and IO_3_^–^ anions are adsorbed on the surface of the HEP, but I^–^ ions show relatively high preference, which is also proved by our XPS measurement of the used photocatalyst (Figure S13a) and is crucial for the Z-scheme water splitting. The adsorption behavior of I^–^ and IO_3_^–^ anions on bare Na_0.56_WO_3–*x*_ was also undertaken (Figure S23). It is obvious that the I^–^ ions are preferentially adsorbed especially at low concentrations. Therefore, the sufficient adsorption of I^–^ on Na_0.56_WO_3–*x*_ enables to accept the photogenerated holes, reducing the recombination with photogenerated electrons and hence promoting the selective H_2_ evolution. However, beyond 1 mM concentration, IO_3_^–^ adsorption is dominated. Hence, in the presence of excess IO_3_^–^ ions, H_2_ evolution is limited, which agrees well with our control experiment (Figure S22). We also found out that only O_2_ is produced in the WOZ with only the IO_3_^–^ mediator (Table 1, run 3), wherein one can see the reason that IO_3_^–^ can relatively easily adsorb on the HEP (Figure 6b) and its reduction strongly competes with proton reduction (Figure S22). Therefore, controlling the concentration of IO_3_^–^ ions in the WOZ is one key issue, which from another angle proves that the initial addition of only I^–^ mediator instead of both I^–^ and IO_3_^–^ mediators is of importance as demonstrated in our experimental design. We also found that continuous production of H_2_ and O_2_ for a prolonged time in our WOZ is stable and reproducible and the concentration of I^–^ and pH were observed to be constant, demonstrating a highly selective and efficient Z-scheme system. A similar experimental study was reported previously^48^ in which the selective adsorption of IO_3_^–^ anion on WO_3_ (OEP) was observed, leading to the selective O_2_ evolution reaction.

To consolidate the adsorption behavior observed experimentally, the redox molecules′ adsorption on photocatalysts was also studied by DFT calculation. The description of the model for WO_3_Pt_4_ and Na_0.625_WO_2.875_Pt_4_ is detailed in the Supporting Information. IO_3_^–^ and I^–^ were adsorbed onto each catalyst at two sites: over the platinum cluster and directly onto the surface of the catalyst. The optimized catalyst models, as well as the adsorption models, can be seen in Figures S24–S31. Adsorption energies are calculated using eq 9, where *E* represents the enthalpy, A is the adsorbent, B is the adsorbate, and AB is the complex structure.
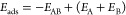
9

**Figure 7:**
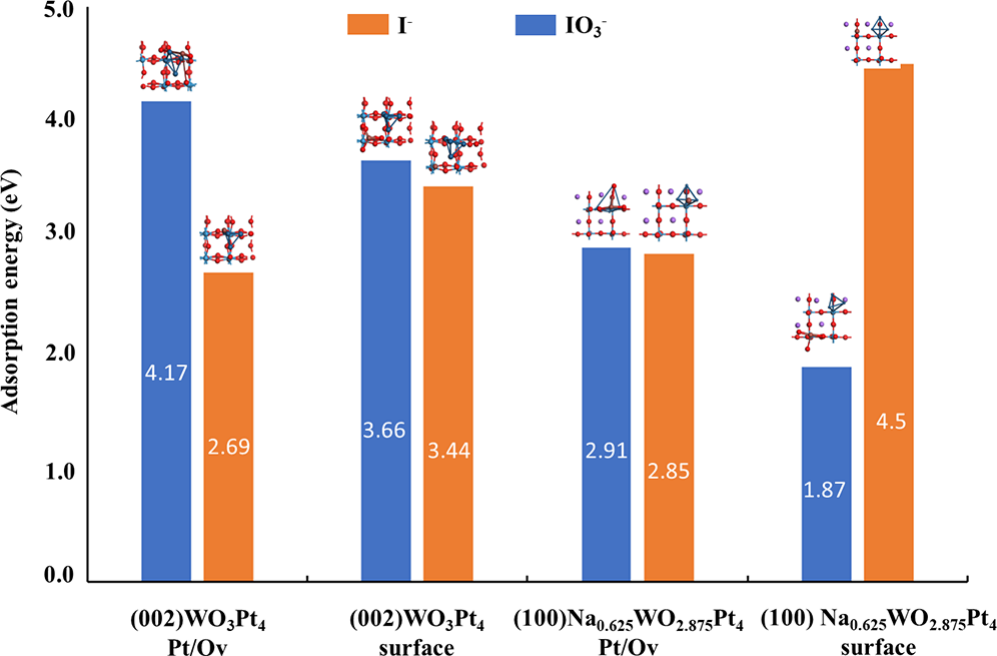
Adsorption energies and selected configurations of IO_3_ and I adsorbed onto the surface of WO_3_Pt_4_ and Na_0.625_WO_2.875_Pt_4_. Pt/Ov represents adsorption onto the Pt cluster, while surface represents adsorption onto the catalyst surface.

The calculated adsorption energies for each tested adsorption site are shown in Figure 7. It can be seen that, for the catalyst WO_3_Pt_4_, iodate is more easily adsorbed than iodide (in good agreement with experimental results). For the Na_0.625_WO_2.875_Pt_4_ catalyst, it was found that both iodate and iodide were adsorbed onto the catalyst; however, iodide was adsorbed to a greater degree onto the catalyst. In each case, iodate adsorbed more preferentially onto the Pt cluster for subsequent reduction reactions, while iodide adsorbed more easily onto the catalyst surface for subsequent oxidation reactions. It should be noted that for both catalysts, the relaxation of iodate led to the stretching of the I=O bonds in every case, even when constraints were applied and, in most cases, the O atom dissociated. Overall, the DFT calculation results suggest that the preferential adsorption of IO_3_^–^ on OEP and I^–^ on HEP would be the reason for the enhanced selective H_2_ and O_2_ evolution using the present Z-scheme.

We also attempted to realize a direct Z-scheme (in the absence of a redox couple, maybe a solid Z-scheme concept) by mixing 3%Pt-Na_0.56_WO_3–*x*_ and 0.5%Pt-WO_3_ powders in an aqueous solution to produce H_2_ and O_2_, simultaneously. However, no gas evolution is noted (Table 1, run 11), suggesting that the simple physical mixing of the HEP and OEP powders cannot work well for water splitting herein, which is also useful for the separation of H_2_ and O_2_ production in two compartments based on the particle suspension Z-scheme rather than solid Z-scheme that produce mixed H_2_ and O_2_ in one cell.

**Scheme 1:**
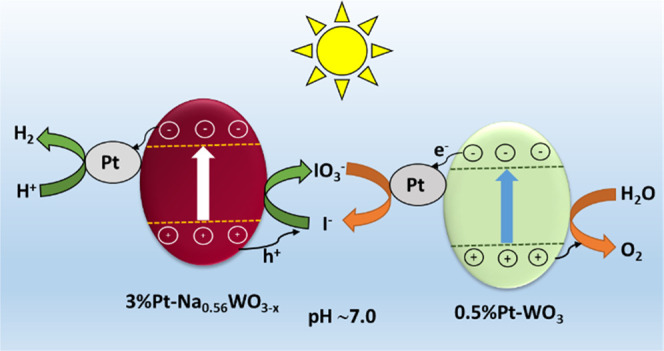
Proposed Reaction Pathway for the New WOZ Water Splitting System

Based on the above results, we proposed the reaction pathway for the new WOZ system schematically in Scheme 1, which is supported by the above investigation. The stoichiometric overall water splitting at pH 7.0 occurs in the presence of a redox mediator IO_3_^–^/I^–^. Upon irradiation, photoexcited electrons from Pt-Na_0.56_WO_3–*x*_ transfer to Pt active sites, where proton reduction occurs to produce H_2_, and the corresponding holes oxidize I^–^ ions into IO_3_^–^, which gets regenerated back to I^–^ by the photogenerated electrons on Pt-WO_3_, while photogenerated holes on Pt-WO_3_ perform water oxidation to generate O_2_, resulting into a complete cycle.

## 3 Conclusions

In summary, we have demonstrated the original tungsten oxides-only suspension Z-scheme for efficient photocatalytic pure water splitting under the full-arc and visible light conditions in the presence of the IO_3_^–^/I^–^ redox couple. Such a design enables separately and readily optimization of each photocatalyst and the cocatalyst. So the stepwise independent H_2_ and O_2_ evolution half-reactions optimization was carried out to minimize/eliminate undesirable reactions. The band position measurements indicate a matched electronic structure for the new Z-scheme. Then, for the first time, the WO_3_-based Z-scheme has been validated for visible-driven complete water splitting by a double excitation mechanism. This novel particulate WOZ exhibits 6.06% AQY at 420 nm when considering the electron number for H_2_ production as 2. Furthermore, I^–^ was found to be favorably adsorbed on Na_0.56_WO_3–*x*_ and IO_3_^–^ on WO_3_ nanosheets by both experimental runs and theoretical modeling, being crucial for an efficient Z-scheme system. The low cost and ensured stability of tungsten oxides are also highly appropriate to subseqent scaling up.

## 4 Experimental Section

### 4.1 Materials and Reagents

Sodium tungstate dihydrate (Na_2_WO_4_·2H_2_O), sodium borohydride (NaBH_4_), sodium iodide (NaI), and sodium iodate (NaIO_3_) were purchased from Sigma Aldrich and used as received. All solutions were prepared using deionized (DI) water. Other chemicals used for experiments were purchased from commercial sources and used without further purification.

### 4.2 Synthesis of HEP

Sodium tungsten oxide bronze (Na*_x_*WO_3–*x*_) was synthesized by reducing the sodium tungstate dihydrate (Na_2_WO_4_·2H_2_O) using sodium borohydride (NaBH_4_).^49^ Briefly, 25 mL of 2.4 M NaBH_4_ prepared in ice-cold water at pH >10 (essential to suppress the hydrogen evolution from NaBH_4_ for upholding the reducing power) was added dropwise to 25 mL of 0.24 M Na_2_WO_4_·2H_2_O solution (pH decreased to 6.5) under stirring. Simultaneously, 5 M HCl was added dropwise to the stirring solution to maintain the pH of ∼6.5 because the addition of NaBH_4_ would raise the pH due to the formation of NaOH and NaBO_2_. This mixture was stirred continuously for 2 h, resulting in a dark brown precipitate, which was kept undisturbed for 2 h for settling down the precipitate. Then, the suspension was centrifuged to separate the precipitate for 10 min at 9000 rpm (each run) and the residue was washed several times with water to remove unreacted precursors, followed by drying at 70 °C for 2 h in a vacuum oven. To obtain crystalline Na*_x_*WO_3–*x*_, the synthesized sample was annealed at 800 °C for 3 h (denoted Na_0.56_WO_3–*x*_) under a N_2_ atmosphere in a tube furnace.

### 4.3 Synthesis of OEP

Two-dimensional (2D) tungstic acid (WO_3_·H_2_O) nanosheets were synthesized as per the earlier report.^40^ Briefly, 120 mM Na_2_WO_4_·2H_2_O was prepared in 40 mL of DI water, and 4 mL of HCl was added in a dropwise manner while stirring for 1 h. The obtained pale green suspension was then centrifuged for 10 min at 9000 rpm (each run) to separate the precipitate, and the residue was washed several times with water to remove the unreacted precursors, followed by drying in an air oven at 70 °C overnight. The as-synthesized WO_3_·H_2_O nanosheets were annealed at 500 °C for 3 h in a muffle furnace at a heating rate of 10 °C min^–1^ to obtain monoclinic WO_3_ nanosheets.

### 4.4 Platinum Cocatalyst Loading

A 3wt%Pt cocatalyst was loaded onto the Na_0.56_WO_3–*x*_ (HEP) surface and 0.5wt%Pt was loaded on WO_3_ nanosheets (OEP) using a photodeposition method by continuous full-arc irradiation of the 10% aqueous methanol solution containing a H_2_PtCl_6_·6H_2_O precursor for 1 h using a 300 W Xe lamp (*TrusTech PLS–SXE 300/300UV)*. Later, the reaction mixture was centrifuged, followed by washing several times to remove the unreacted Pt precursors. Then, the residue was dried at 80 °C for 12 h under vacuum. The HEP photocatalyst was immediately used after the centrifugation.

### 4.5 Characterization

The X-ray diffraction (XRD) patterns were obtained using a STOE STADI-P diffractometer with Mo Kα as the X-ray radiation source. UV–Vis diffuse reflectance spectra (DRS) of the HEP and OEP powders were collected using an Agilent Cary 5000 spectrophotometer fitted with an integrating sphere using standard barium sulfate powder as a reference. TEM measurements were performed using a JEOL2100 TEM, operated at 200 kV. Samples for TEM measurements were prepared by drop-casting the dilute dispersions of the photocatalysts onto the carbon-coated copper grids. X-ray photoelectron spectroscopy (XPS) measurements were undertaken using a Kratos Axis SUPRA machine using monochromated Al-Kα irradiation as a source of X-rays. XPS data analysis was performed using Casa XPS software. Shirley/Touguard methods were used for background corrections. Raman spectra were measured on a Renishaw inVia Raman microscope, using a 532 nm excitation laser. The photoelectrochemical onset potential measurements were recorded using linear sweep voltammetry by sweeping the potential from +0.8 to −0.5 V vs Ag/AgCl in a conventional three-electrode (photocatalyst film-deposited electrode as the working electrode, Ag/AgCl as the reference electrode, and a platinum mesh as the counter electrode) cell using an electrochemical analyzer (IVIUM Technologies). A 0.1 M Na_2_SO_4_ (pH ∼7.0) solution was used as the electrolyte in the presence of 10% methanol as a hole scavenger. The photocatalyst film was prepared by dispersing 20 mg of the photocatalyst in a 5.4 mL solution comprising water:ethanol at a 4:1 (v/v) ratio and 0.4 mL of Nafion (5% solution), followed by 1 h ultrasonication. 50 μL of the obtained slurry was drop-cast onto the precleaned fluorine-doped tin oxide (FTO) conducting substrate, followed by drying at 60 °C in an oven before the electrochemical tests. The area of the photocatalyst thin film was 1 × 1 cm^2^, whereas the size of the FTO substrate was 1 × 2 cm^2^. The catalyst loading amount was ca. 3.7 mg mL^–1^.

### 4.6 Photocatalytic Studies

The H_2_ and O_2_ evolution half-reactions and their simultaneous production in a Z-scheme system were carried out in a custom-made glass batch reactor with a top quartz window. The known amount of photocatalysts was loaded in the reactor containing 70 mL of water and 5 mM NaI and dispersed well by ultrasonication for 30 min. The pH of the solution was controlled by adding dilute solutions of H_2_SO_4_ and NaOH. The reactor was sealed and purged with high-purity Ar gas for 1 h to remove air/dissolved oxygen in the solution and headspace. After baseline measurement (0 h), the reactor was irradiated using a 300 W Xe lamp (Newport). The reactor was placed in a water bath during irradiation to maintain the reaction temperature. The production of H_2_ and O_2_ gases was quantified at regular intervals using gas chromatography (Varian 430-GC, TCD, argon carrier gas) equipped with a molecular 5A column using Ar as the carrier gas and N_2_ as the internal reference. The apparent quantum yield (AQY) was determined by performing photocatalytic experiments at specific wavelengths by using an appropriate bandpass filter. For AQY measurements, 60 mg of HEP and 30 mg of OEP were dispersed in 70 mL of water containing 5 mM NaI. The reactor was irradiated through a 2 cm diameter aperture, enabling the central beam through, which is very reliable for the AQY analysis as highlighted by Domen et al.^50^ The light intensity of the lamp was measured at five different points to obtain an average intensity using a calibrated photodiode coupled with an optical power meter (Newport, Model 1908-R). The measured light intensity with a 420 nm bandpass filter was 0.5 mW cm^–2^. The AQY was calculated (see Supporting Information for detailed calculation) using the following equation
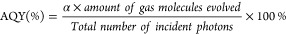
10where α = 2 and 4 for H_2_ and O_2_ evolution reactions provided that there are 2 electrons required for one H_2_ molecule production and 4 holes for one O_2_ molecule production, respectively. The GC calibration curves for H_2_ and O_2_ are provided in Figure S32. Furthermore, the raw GC chromatograms for the Z-scheme water splitting, under a full-arc condition, corresponding to the gas evolution shown in Figure 5a, are given in Figure S33.

### 4.7 Adsorption Study

The adsorption of I^–^ and IO_3_^–^ ions on the surface of the HEP and OEP was studied by measuring their concentrations using UV–Vis absorption spectroscopy. The determination of the I^–^ ion concentration is straightforward as it can show the absorption peak at 225 nm without adding any reagent. However, IO_3_^–^ shows absorption peaks at 288 and 352 nm only in the presence of an excess of I^–^ and 0.2 M H_2_SO_4_. The calibration curves for I^–^ and IO_3_^–^ are shown in Figures S34 and S35, respectively. We monitored the peak at 352 nm to determine the concentration of IO_3_^–^.

### 4.8 Computational Details

Density functional theory (DFT) calculations were carried out to investigate the adsorption of IO_3_^–^ and I^–^ onto both WO_3_Pt_4_ and Na_0.625_WO_2.875_Pt_4_. The structure of Na_0.625_WO_2.875_Pt_4_, through XRD analysis, was deemed to be structurally similar enough to the experimentally investigated Na_0.56_WO_3–*x*_. For the adsorption calculations, BIOVIA Materials Studio (MS) package was used with the generalized gradient approximation (GGA) and Perdew–Burke–Ernzerhof (PBE) functional to account for the exchange-correlation energy.^51^ All calculations were spin-polarized, with the projector augmented wave (PAW) pseudopotentials and the Van der Waals interactions were described by the Grimme DFT-D3 method.^52^ The Kohn–Sham equations were solved with a convergence criterion of 2.0e-5 eV/atom for energy and 0.05 eV Å^–1^. Additionally, *k*-point sampling was carried out using the Monkhorst–Pack scheme with a (1 ×1 × 1) grid, and a cutoff energy of 489.8 eV was used.

## Supporting Information Available

Structural representation of materials; SEM, TEM, and EDX of HEP and OEP; XRD, and XPS data before and after photocatalysis; UV–Vis of HEP and OEP; photoelectrochemical onset potential measurements; Mott–Schottky measurements; pH optimization; H_2_ evolution activity at longer wavelengths; control experiments; AQY comparison with optical spectra of HEP and OEP; DFT results; GC calibration and raw data; and adsorption of I^–^ and IO_3_^–^ (PDF)
