# Supplementary material for: Tungsten Oxide-Based Z-Scheme for Visible Light-Driven Hydrogen Production from Water Splitting
Source: ACS Catal. 2023 Jun 26;13(13):9113–24. doi: 10.1021/acscatal.3c01312 (PMC10334426; doi:10.1021/acscatal.3c01312)
Supplement: Supplementary file 1 — cs3c01312_si_001.pdf [file cs3c01312_si_001.pdf]

Supporting Information

Tungsten oxide-based Z-scheme for visible light-driven hydrogen production from water splitting

Madasamy Thangamuthu[1], Kiran Vankayala[1], Lunqiao Xiong, Stuart Conroy, Xiaolei Zhang, Junwang Tang**

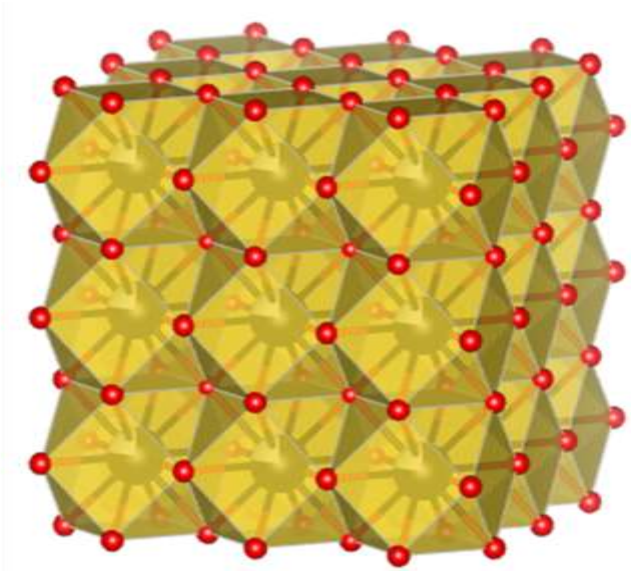

Figure S1. Structural representation of cubic Na_xWO_{3-x}

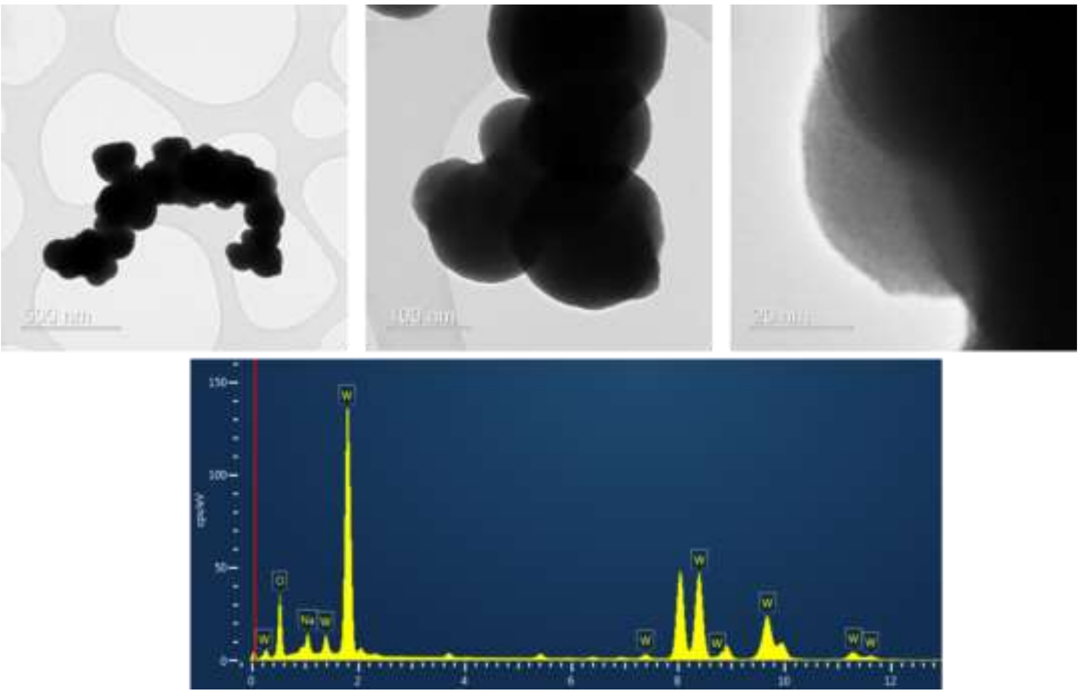

Figure S2. TEM images of the as-synthesized Na_xWO_{3-x} recorded under different magnifications and the corresponding EDX spectrum.

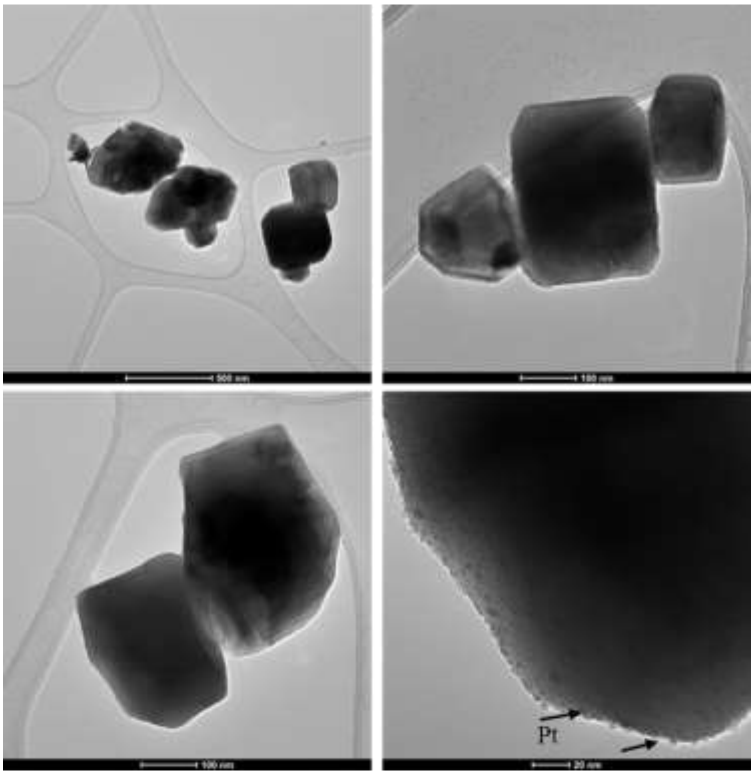

Figure S3. TEM images of Pt-$Na_{0.56}WO_{3-x}$ recorded under different magnifications. The presence of Pt nanoparticles on the surface is visible (indicated with arrows).

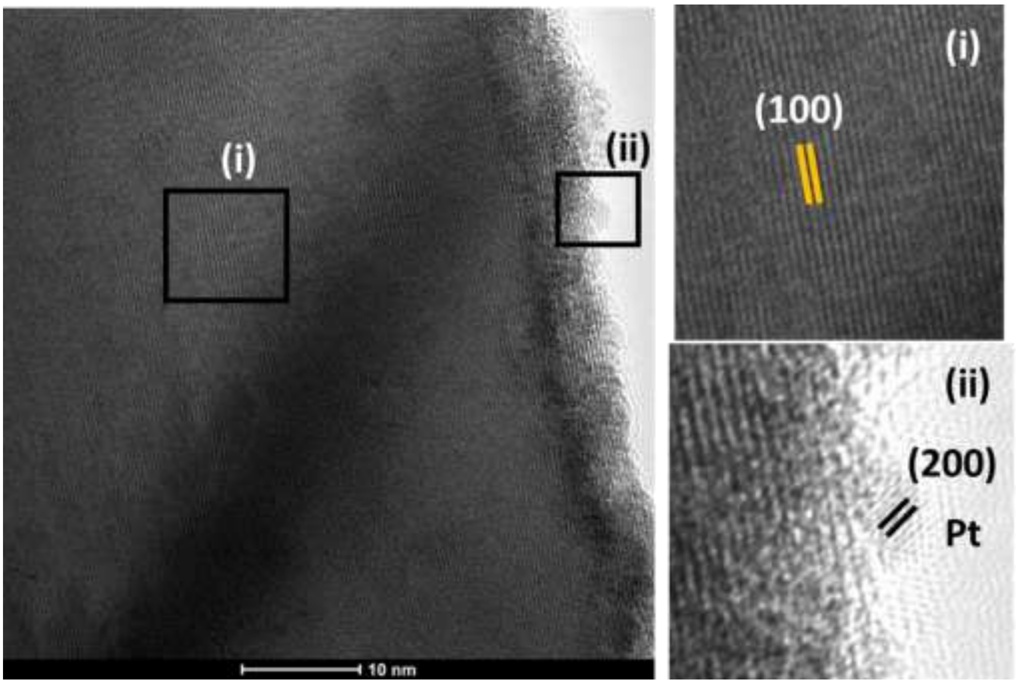

Figure S4. HRTEM image of Pt-$Na_{0.56}WO_{3-x}$. The amplified regions marked with box areas are shown in (i) and (ii). (i) represent regions of $Na_{0.56}WO_{3-x}$ and (ii) represent Pt nanoparticles.

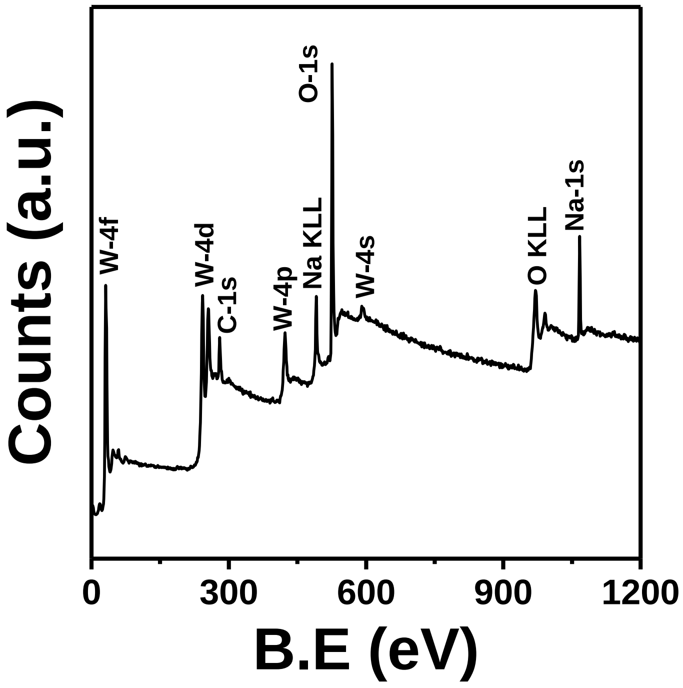

Figure S5. XPS survey spectra of $Na_{0.56}WO_{3-x}$

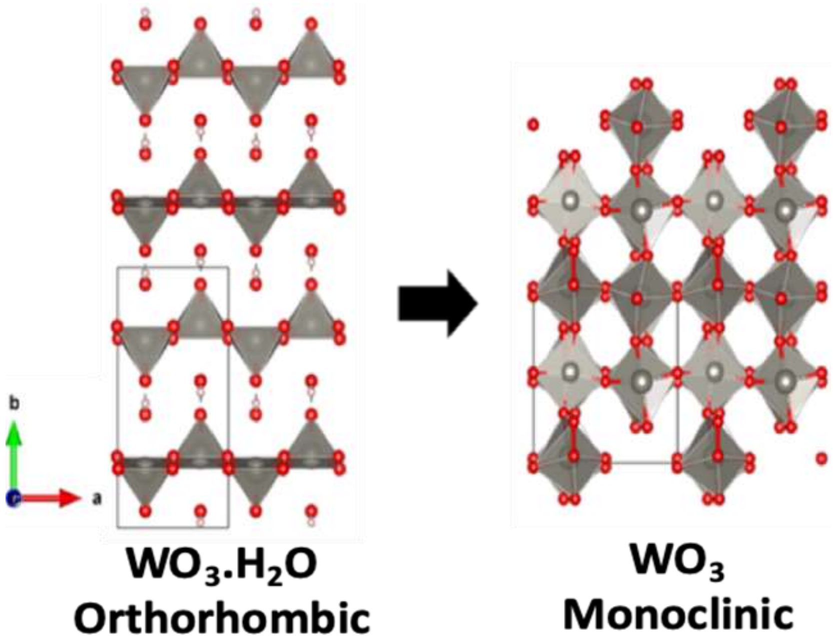

Figure S6. Structural representation of orthorhombic $WO_3{\cdot}H_2O$ and monoclinic WO_3 nanosheets.

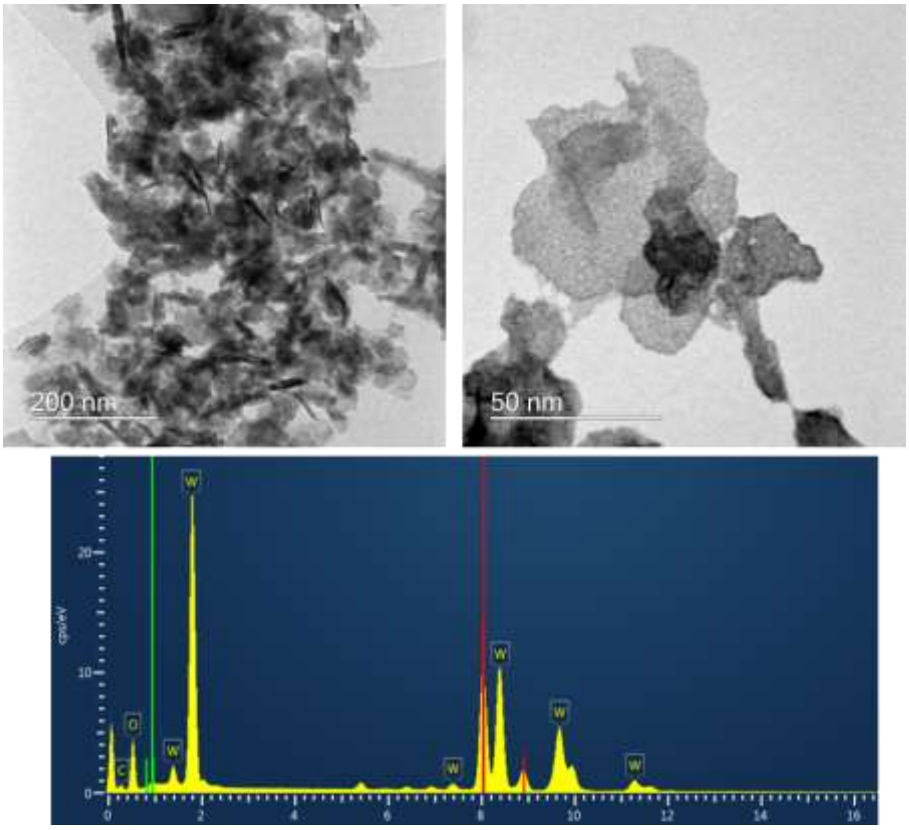

Figure S7. TEM images of the as-synthesized $WO_3.H_2O$ nanosheets recorded under different magnifications and the corresponding EDX spectrum.

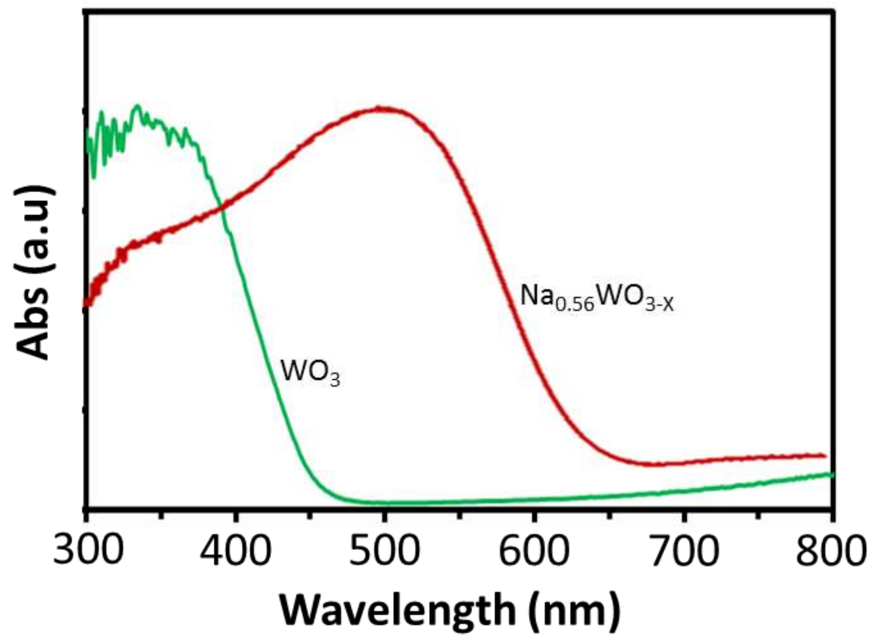

Figure S8. UV-Vis absorbance spectra of WO_3 nanosheets, and $Na_{0.56}WO_{3-x}$.

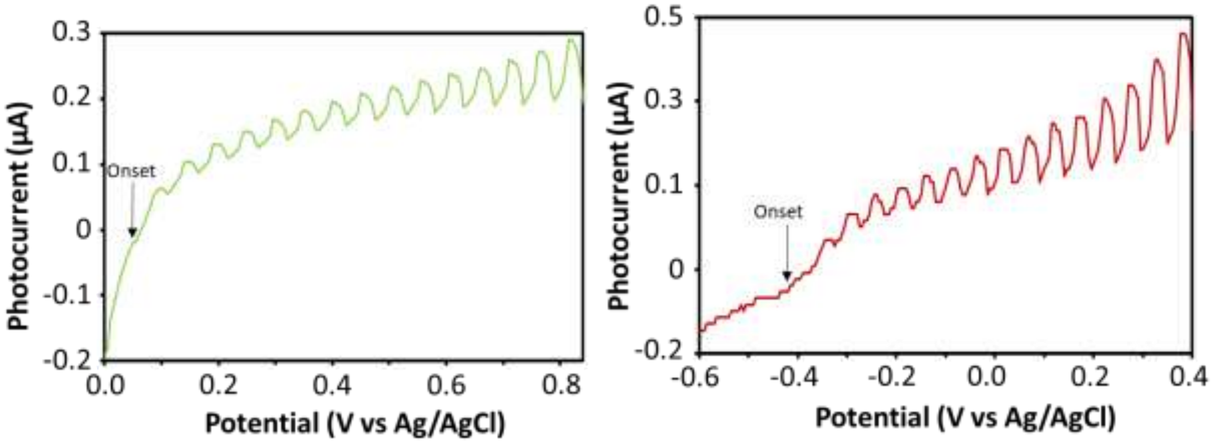

Figure S9. a and b are the photocurrent onset potential observed for WO_3 and $Na_{0.56}WO_{3-x}$ in 0.1 M Na_2SO_4 electrolyte at pH 7.0 in the presence of methanol hole-scavenger.

The obtained photocurrent onset potential measurements data were converted to SHE scale using the following equation

$$E_{(SHE)} = E_{Ag/AgCl} + E°_{Ag/AgCl}$$

CB potential calculation for WO_3

$E_{(SHE)} = 0.0 + 0.197 = +0.197$ V

CB potential calculation for $Na_{0.56}WO_{3-x}$

$E_{(SHE)} = -0.4 + 0.197 = -0.203$ V

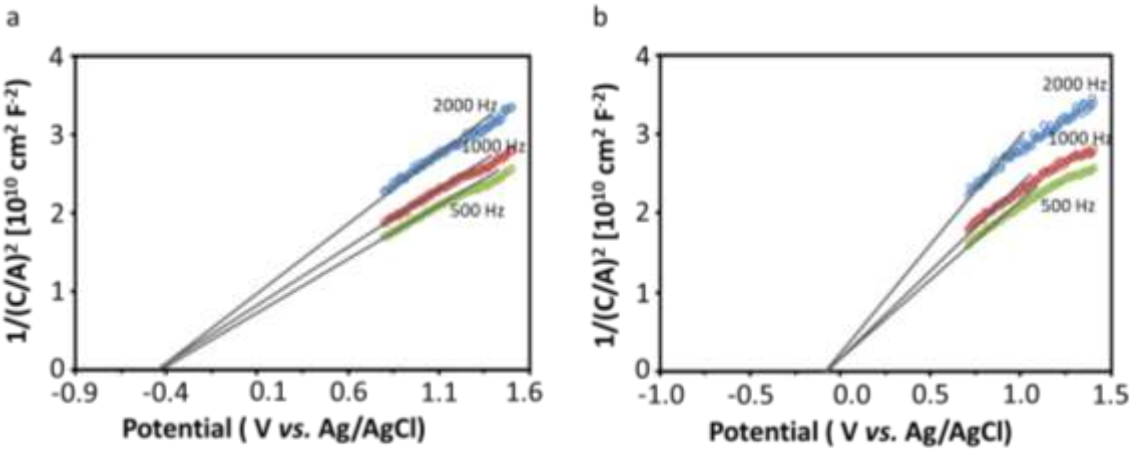

Figure S10. **a** and **b** are the Mott-Schottky plots of the $Na_{0.56}WO_{3-x}$, and WO_3 nanosheets electrodes, respectively measured in 0.1 M Na_2SO_4 (pH 7.0) electrolyte under dark condition.

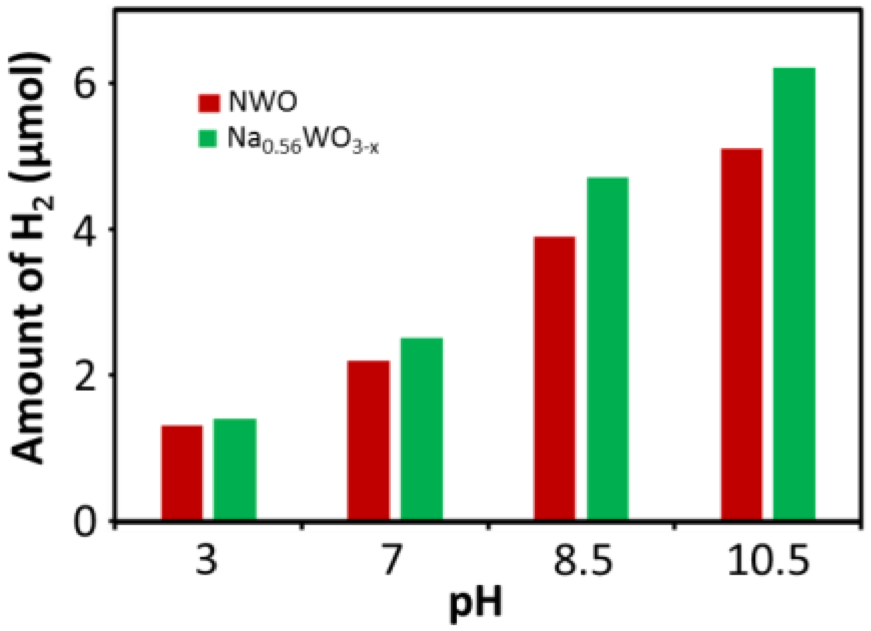

Figure S11. Optimization of pH process. H_2 evolution from water using 20 mg of NWO and $Na_{0.56}WO_{3-x}$ containing 5 mM NaI as a hole-scavenger under full arc condition. The rate of H_2 evolution was obtained from the 6 h reaction.

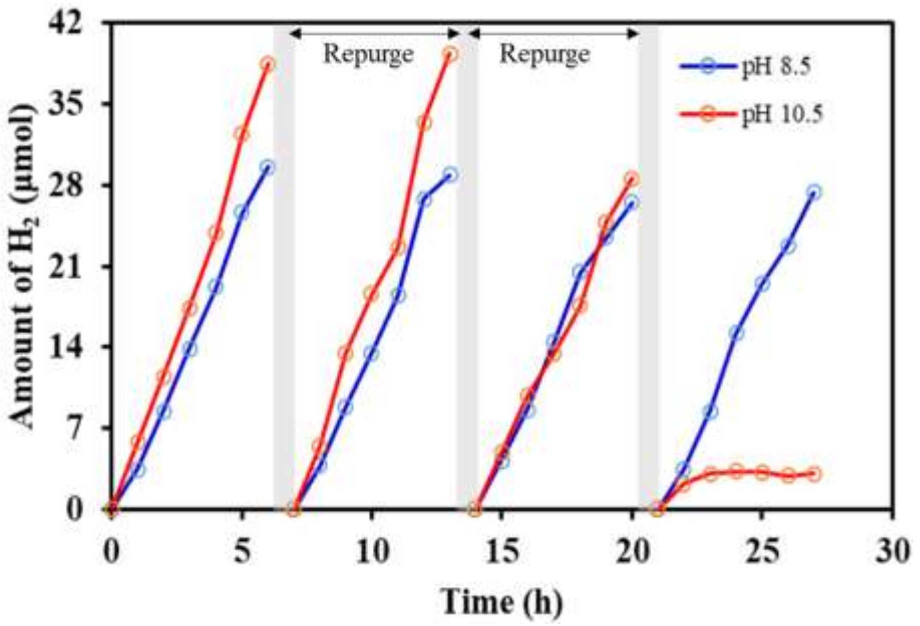

Figure S12. The H_2 evolution stability study on $Na_{0.56}WO_{3-x}$ in an aqueous solution at pH 8.5 and pH 10.5 containing 5 mM NaI under full arc condition for four consecutive 6 hour runs.

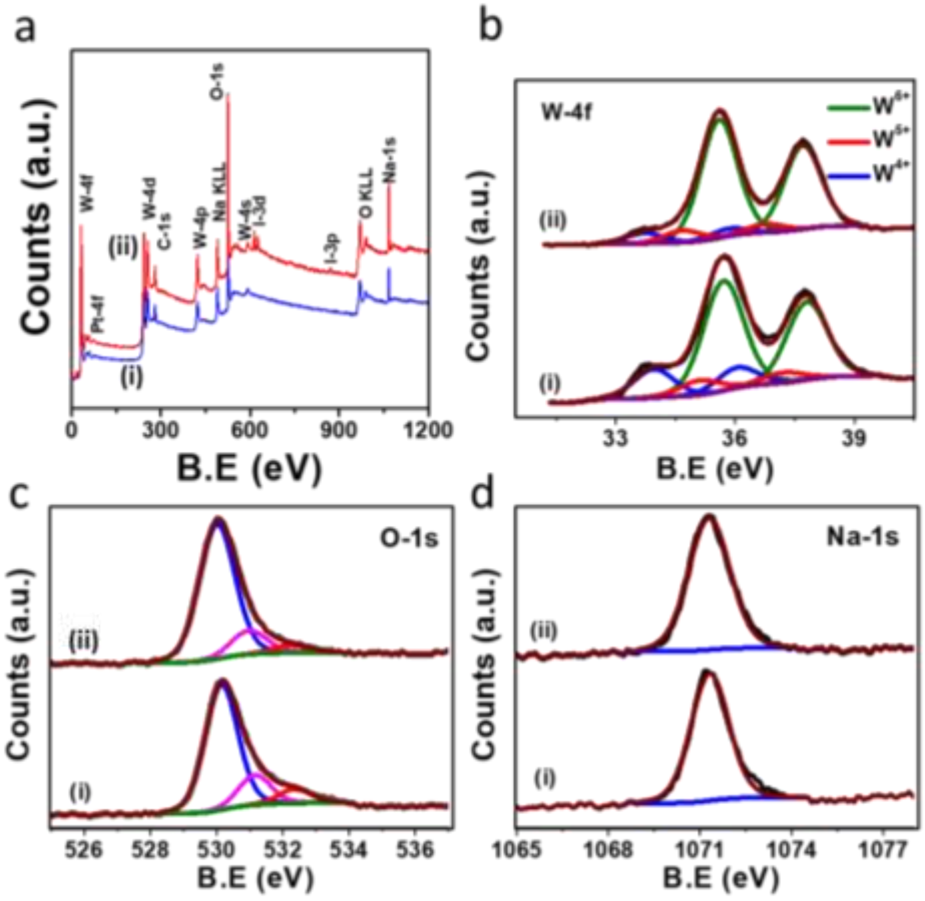

Figure S13. XPS survey and deconvoluted spectra of W-4f, O-1s, and Na-1s regions for 3Pt-$Na_{0.56}WO_{3-x}$ (i) before and (ii) after photocatalysis.

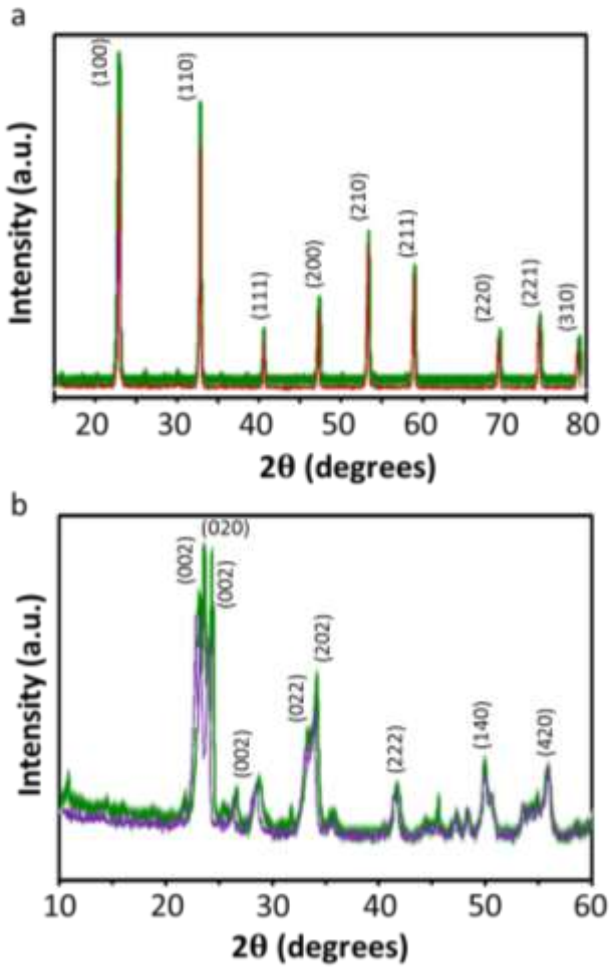

Figure S14. a XRD patterns of the $Na_{0.56}WO_{3-x}$ before (green), and after photocatalysis (red), respectively. **b** XRD patterns of the WO_3 before (green) and after photocatalysis (purple), respectively.

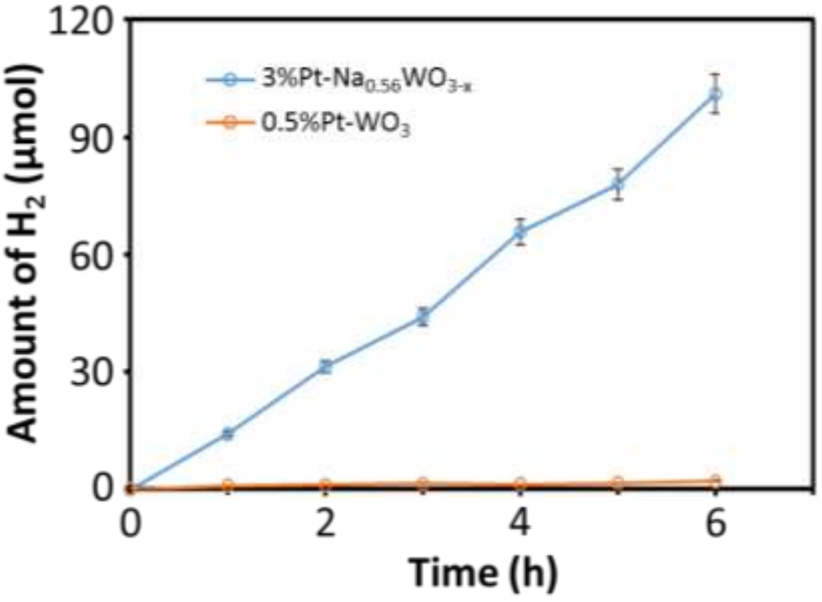

Figure S15. The H_2 evolution activity of the 3%Pt- $Na_{0.56}WO_{3-x}$ and 0.5%Pt-WO_3 nanosheets measured in water (pH = 7.0) containing 5 mM NaI, as a hole scavenger.

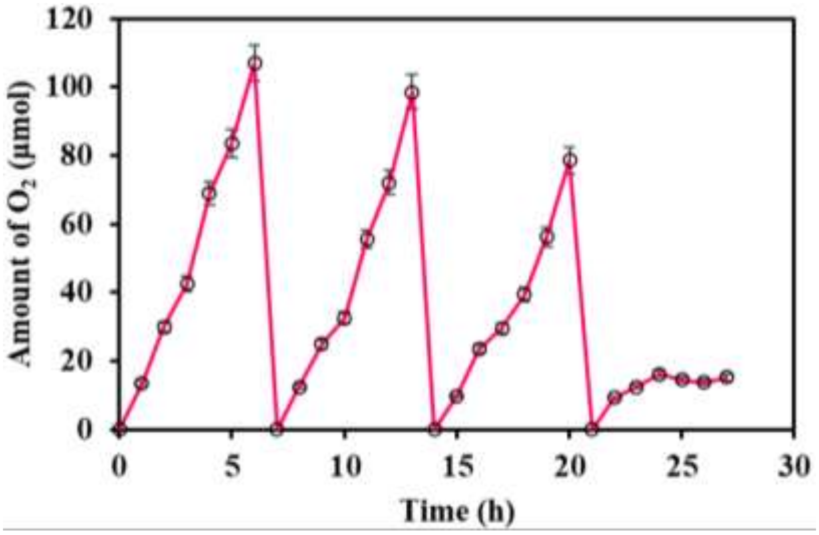

Figure S16. The O_2 evolution stability study on WO_3 in an aqueous solution at pH 10.5 containing 5 mM $NaIO_3$ under full arc condition for four consecutive 6 hour runs, Ar gas was purged after each run.

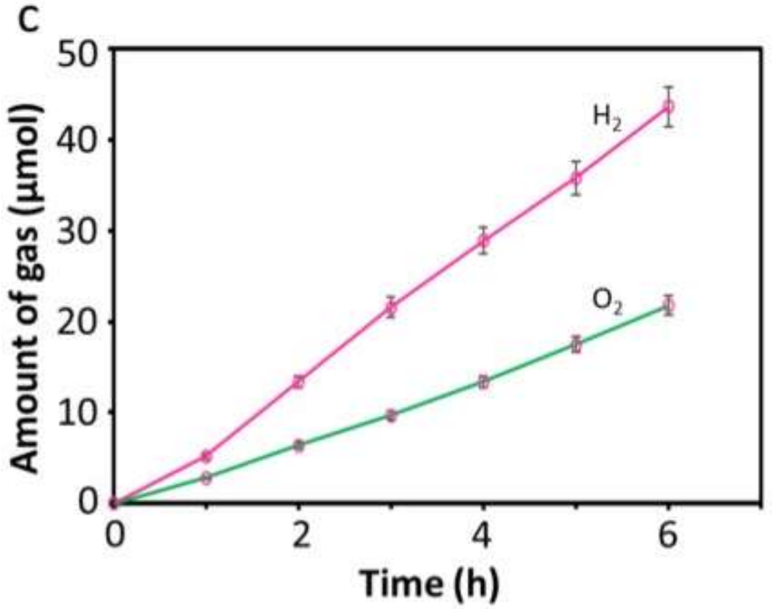

Figure S17. WOZ water splitting activity under visible light irradiation ($\lambda \geq 420$ nm) at pH 7, 1:1 weight ratio of HEP: OEP, 5 mM NaI as redox mediator.

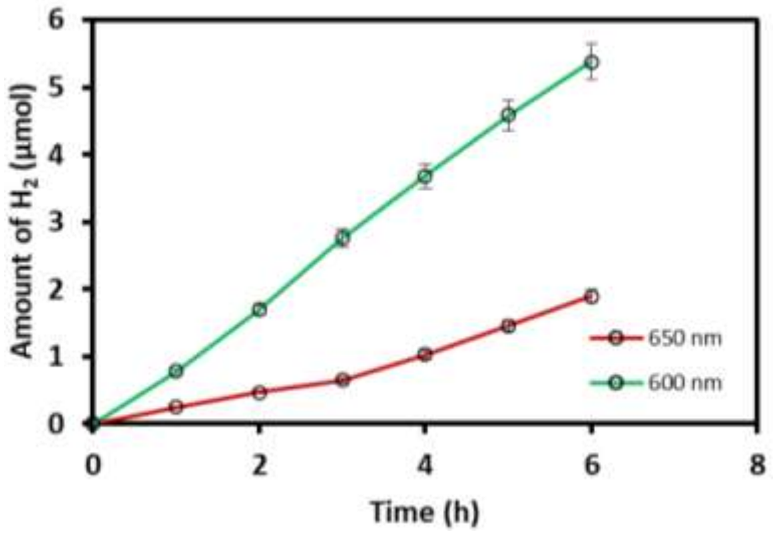

Figure S18. The H_2 evolution activity of the 3%Pt-$Na_{0.56}WO_{3-x}$ under 600 nm and 650 nm monochromatic wavelength irradiation measured in water (pH = 7.0) containing 5 mM NaI, as a hole scavenger.

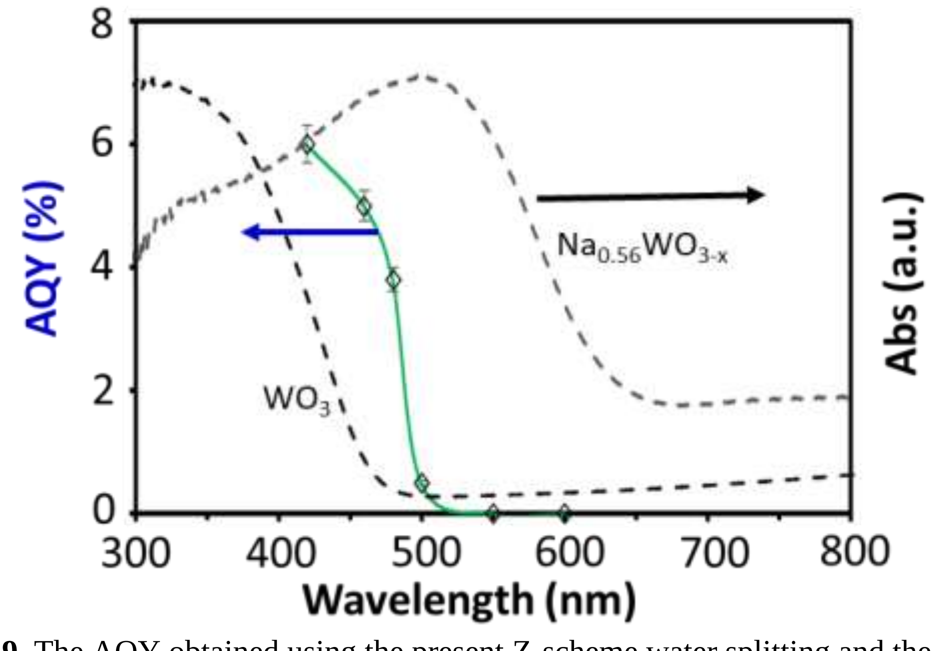

Figure S19. The AQY obtained using the present Z-scheme water splitting and the absorption spectra of HEP and OEP.

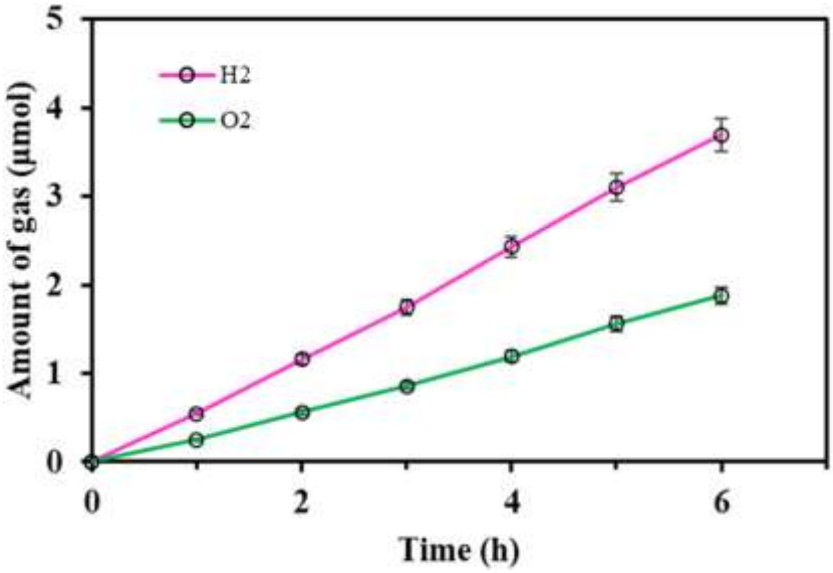

Figure S20. WOZ water splitting system composed of 3wt%Pt-$Na_{0.56}WO_{3-x}$ as the HEP and 0.5wt%Pt-WO_3 nanosheets as the OEP under 420 nm monochromatic irradiation at pH 7.0, 5 mM NaI as redox mediator.

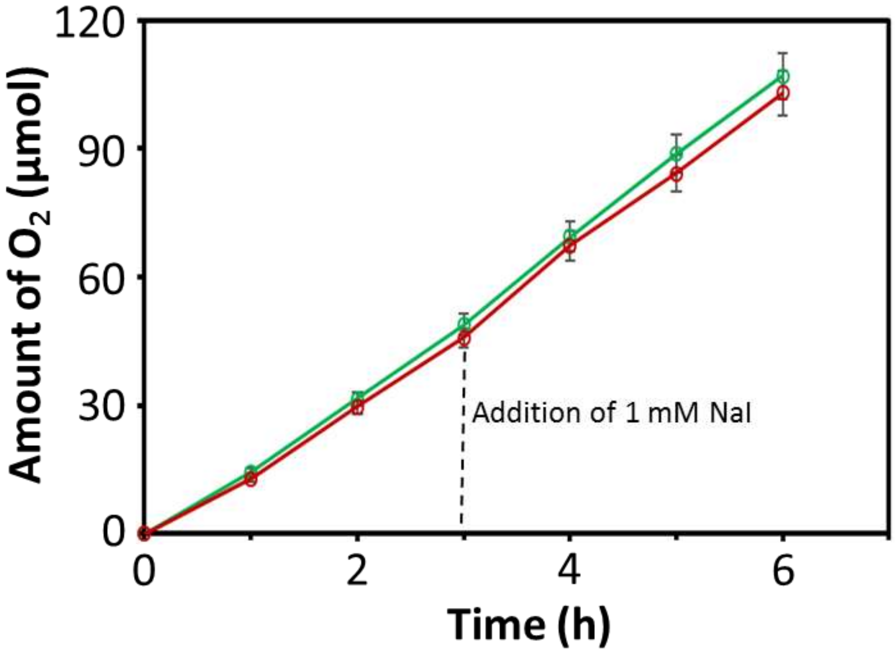

Figure S21. A study on the competitive reaction between I^- oxidation and water oxidation. The addition of 1 mM NaI at 3 h does not change the O_2 evolution activity of 0.5%Pt-WO_3 nanosheets (red) compared to the water oxidation reaction only in the presence of $NaIO_3$ (green).

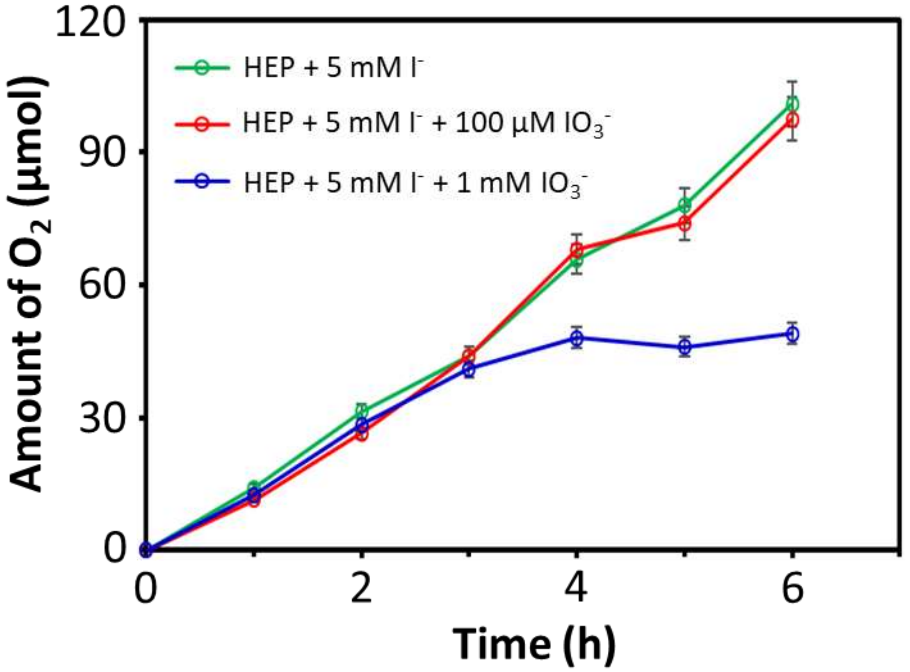

Figure S22. The competitive reaction between water reduction and IO_3^- reduction. H_2 evolution from water (pH 7.0) using 10 mg of 3wt%Pt-$Na_{0.56}WO_{3-x}$ containing 5 mM NaI as a hole scavenger, and the external addition of 100 µM and 1 mM of IO_3^- at 3 h under full arc condition for 6 h.

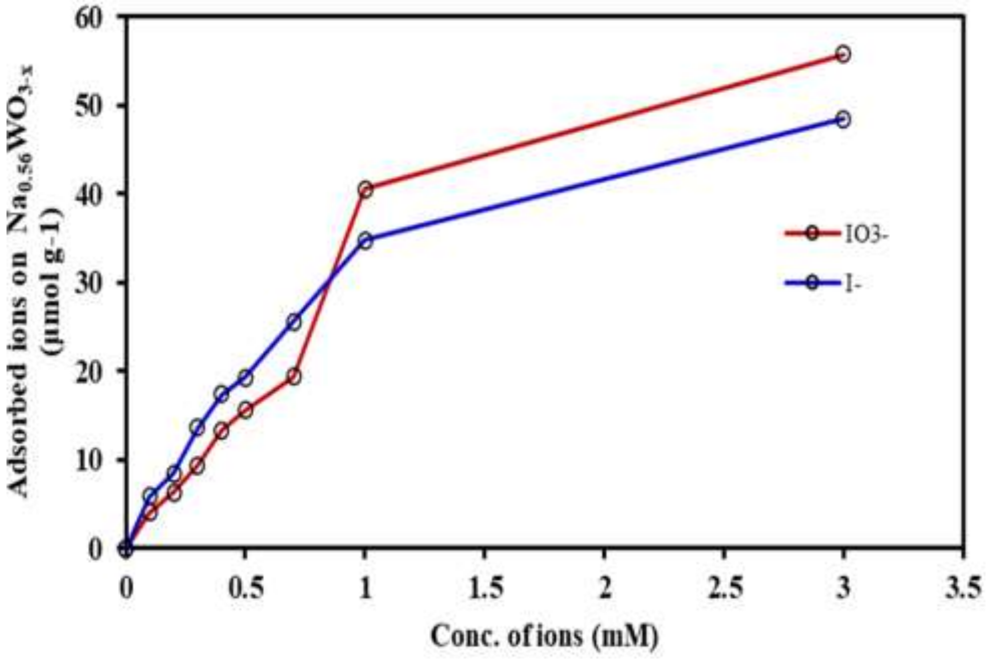

Figure S23. Adsorption behaviour of IO_3^- and I^- anions on the surface of $Na_{0.56}WO_{3-x}$ powder suspended in an aqueous solution at pH ~7.0 under dark conditions.

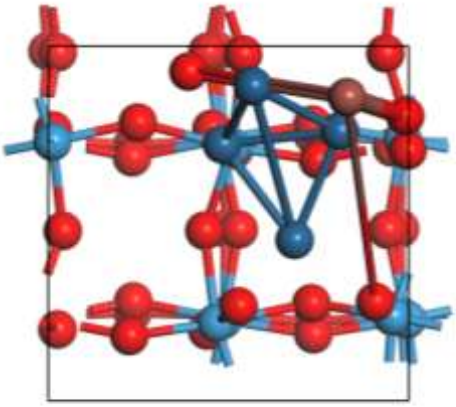

Figure S24. IO_3^- adsorbed onto the Pt cluster atop WO_3. Colour scheme: Brown = iodine.

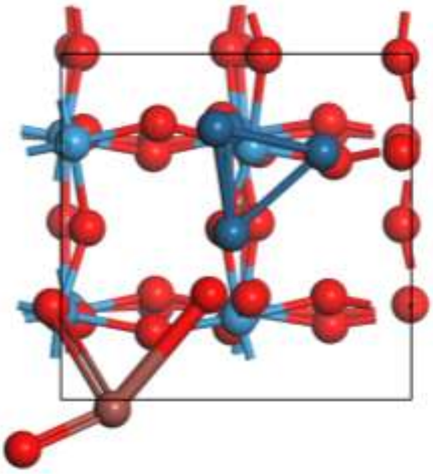

Figure S25. IO_3^- adsorbed onto the catalyst surface of WO_3.

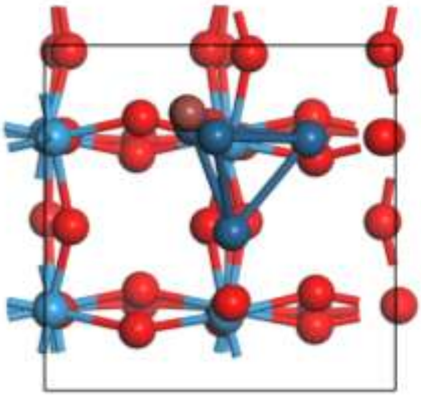

Figure S26. I^- adsorbed onto the Pt cluster atop WO_3.

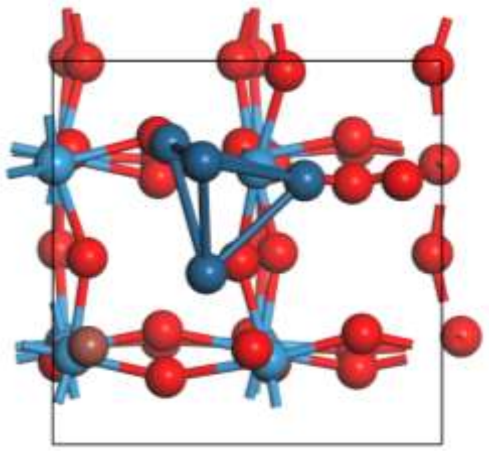

Figure S27. I^- adsorbed onto the catalyst surface of WO_3.

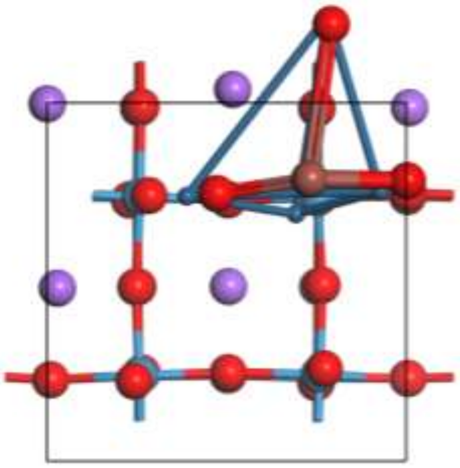

Figure S28. IO_3^- adsorbed onto the Pt cluster atop $Na_{0.625}WO_{2.875}$.

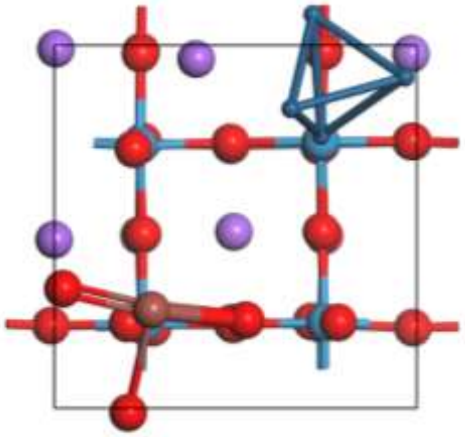

Figure S29. IO_3^- adsorbed onto the catalyst surface of $Na_{0.625}WO_{2.875}Pt_4$.

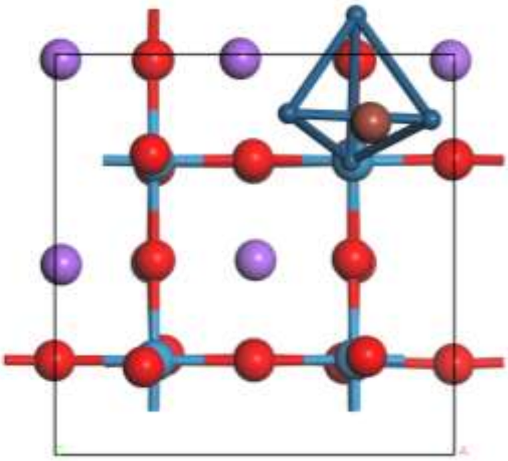

Figure S30. I^- adsorbed onto the Pt cluster atop $Na_{0.625}WO_{2.875}$.

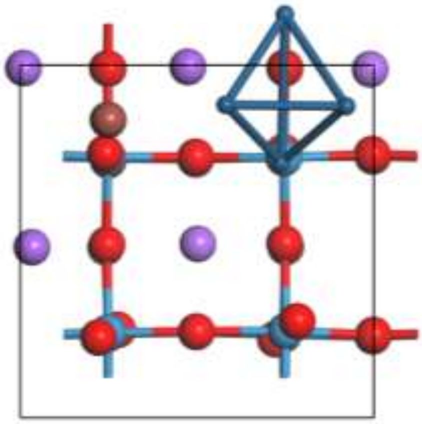

Figure S31. I^- adsorbed onto the catalyst surface of $Na_{0.625}WO_{2.875}Pt_4$.

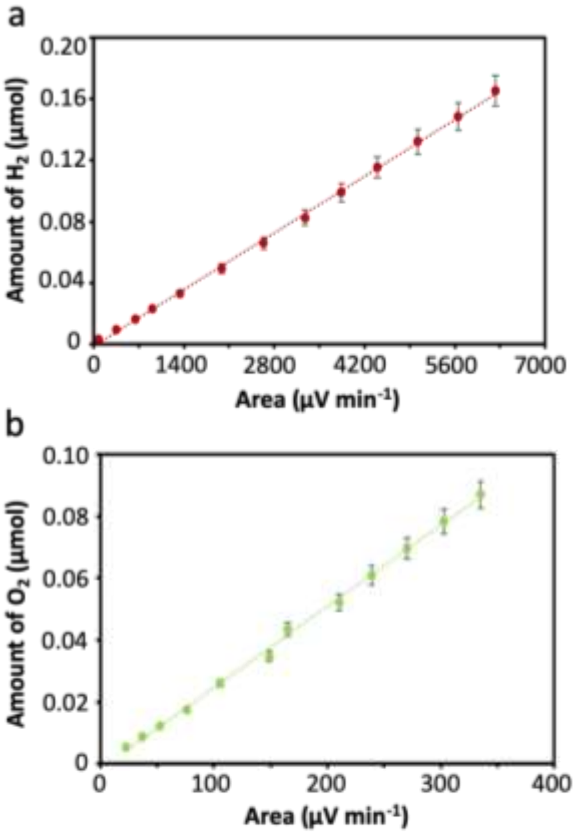

Figure S32. The standard calibration curves for a) H_2, and b) O_2

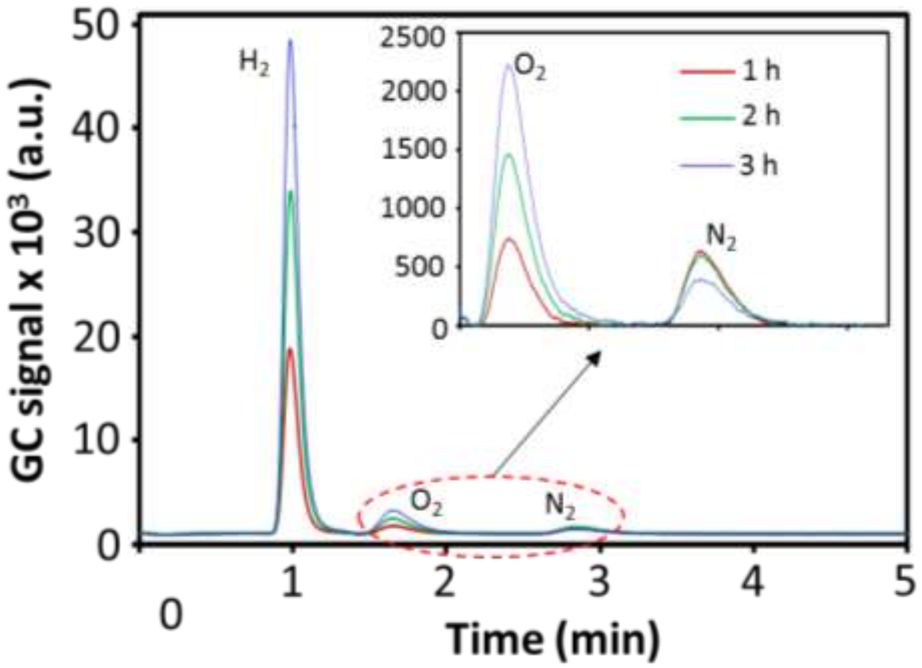

Figure S33. The original GC data obtained for Z-scheme water splitting using 10 mg 3wt%Pt-$Na_{0.56}WO_{3-x}$ and 10 mg 0.5wt%Pt-WO_3 in pure water containing 5 mM NaI at pH 7.0. The zoomed part represents the O_2 evolution.

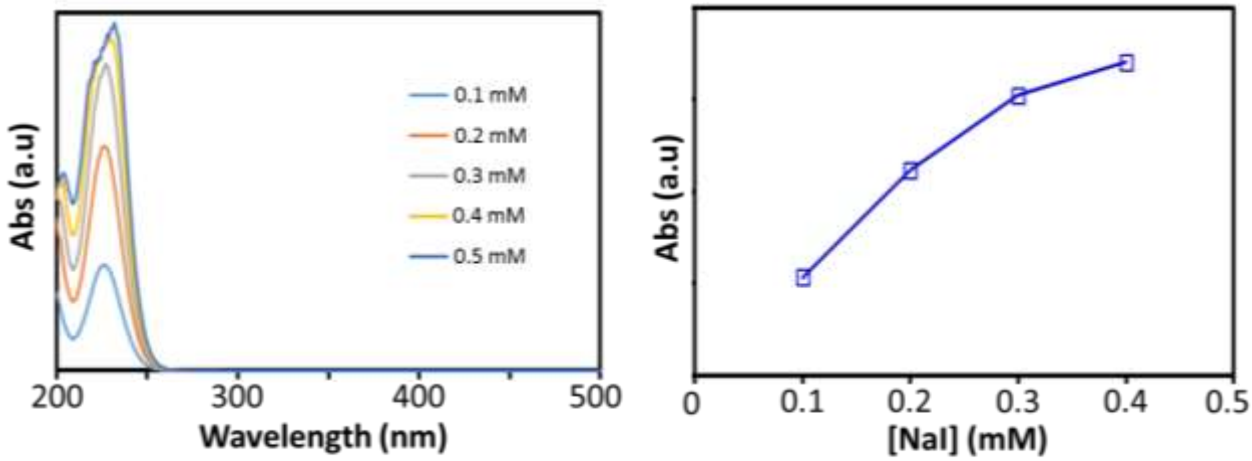

Figure S34. Linear calibration study for NaI. The 60 mg of the 3%Pt-$Na_{0.56}WO_{3-x}$ was loaded in the reactor containing 70 mL of water (pH 7.0) with various concentrations of NaI ranging from 0.1 mM to 0.5 mM.

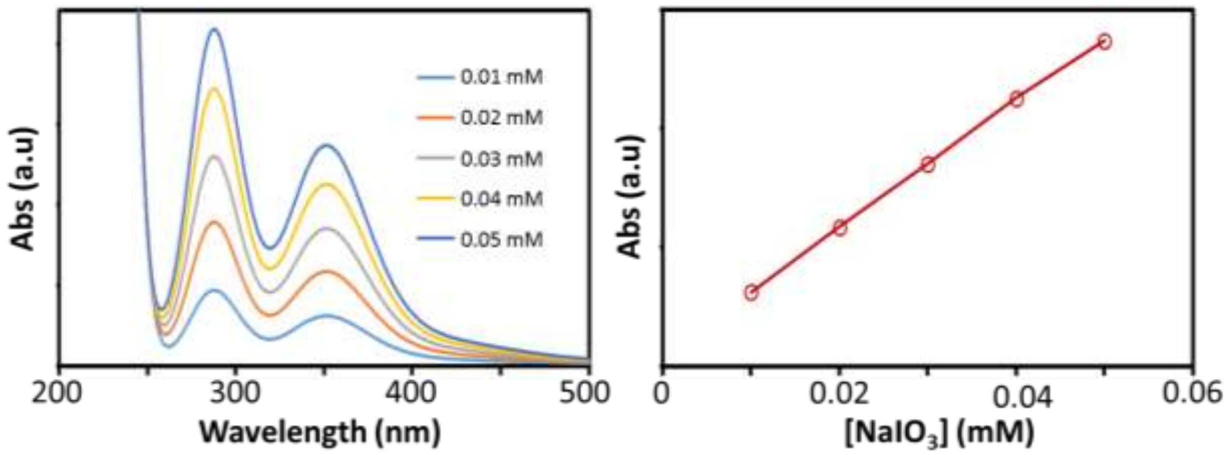

Figure S35. Linear calibration study for $NaIO_3$. The 30 mg of the Pt-WO_3 was loaded in the reactor containing 70 mL of water (pH 8.5) with various concentrations of $NaIO_3$ ranging from 0.01 mM to 0.05 mM.

Description of Models

WO_3Pt_4

For the conventional cell, a WO_3 crystal with a monoclinic space group (P21/n) was imported and the structure relaxed. The optimised cell had lattice parameters a, b, c = 7.448, 7.334, 7.743 Å. The surface was then cleaved to form a (002) surface and a vacuum slab of 15 Å was created to prevent slab-slab interactions perpendicular to the surface. A four-atom Pt cluster was then adsorbed onto the surface of the catalyst to complete the structure (**Figure S36**).

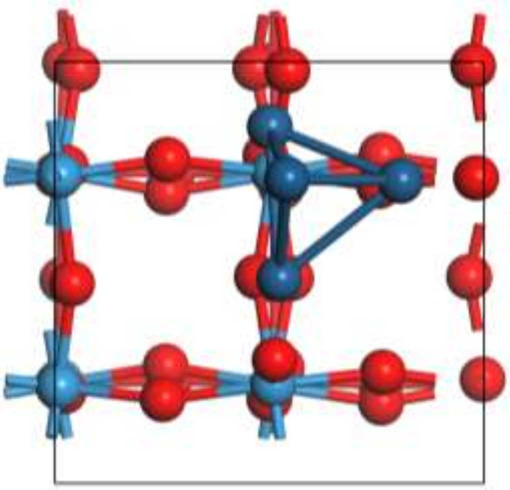

Figure S36. (002) Optimised WO_3Pt_4 catalyst. Colour scheme: red = O, light blue = w, dark blue = Pt.

$Na_{0.625}WO_{2.875}Pt_4$

For the sodium-tungsten bronze, the conventional cell coordinates were taken from literature to create a catalyst as close to the experimentally investigated structure as possible, within our computational constraints.[1] The conventional cell has a cubic space group (Pm-3m) and produces the molecular formula $Na_{0.625}WO_3$, a single O atom on the surface was then removed to form an oxygen vacancy (Ov) and produce the formula $Na_{0.625}WO_{2.875}$. This structure, through XRD analysis, was deemed to be structurally similar enough to the experimentally investigated $Na_{0.56}WO_{3-x}$. In order to select which O atom to remove, three positions were defined which are chemically non-equivalent (**Figure S37**). It was found that the O atom on the top of the surface (Ov3) was the most favourable to be removed and is used herein. The Na0.625WO2.875 was cleaved to form a (100) surface and a vacuum slab of 15 Å was created. A four-atom Pt cluster was then adsorbed onto the surface at various points, with the position over the Ov being the most favourable (**Figure S38**).

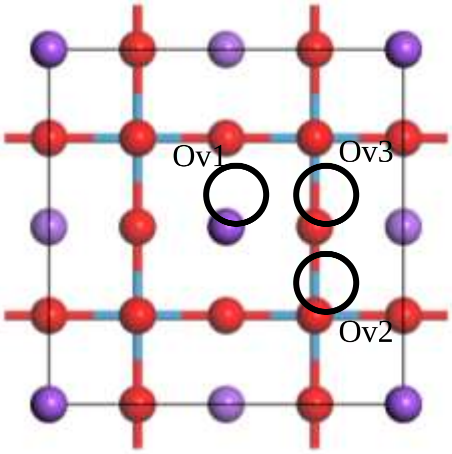

Figure S37. Optimised (100) $Na_{0.625}WO_{2.875}$ structure showing chemically unequivocal O atoms, labelled Ov1, Ov2 and Ov3. Colour scheme: Purple = Na.

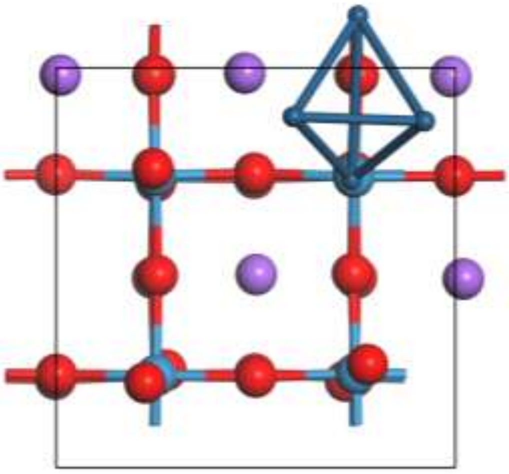

Figure S38. Optimised (100) $Na_{0.625}WO_{2.875}Pt_4$ catalyst structure.

Table S1. The positions of binding energy values of different core levels of $Na_{0.58}WO_3$, Pt-$Na_{0.58}WO_3$ before and after photocatalysis. O_L, O_V, and O_c represent lattice oxygen, oxygen vacancy, and O_c, chemisorbed oxygen, respectively.

Sample	W-4f						O-1s			Na-1s (eV)
	W^{4+}		W^{5+}		W^{6+}					
	$4f_{7/2}$ (eV)	$4f_{5/2}$ (eV)	$4f_{7/2}$ (eV)	$4f_{5/2}$ (eV)	$4f_{7/2}$ (eV)	$4f_{5/2}$ (eV)	O_L (eV)	O_V (eV)	O_c (eV)	
**$Na_{0.58}WO_3$**	33.3	35.4	34.8	36.9	35.5	37.6	530.4	531.6	533.1	1071.2
**Pt-$Na_{0.58}WO_3$ before**	33.9	36.0	35.1	37.2	35.7	37.8	530.1	531.2	532.4	1071.3
**Pt-$Na_{0.58}WO_3$ after**	33.8	35.9	34.7	36.8	35.6	37.7	530.05	531.1	532.3	1071.3

Table S2. Comparison of the photocatalytic activities of various particulate suspension Z-scheme systems for water splitting, which can be potentially operated in a dual bed flow system.

HEP	OEP	Mediators	Irradiation condition	AQY (%) at 420 nm (2 electron process)	Ref.
Pt-$Sm_2Ti_2S_2O_5$	TiO_2(rutile)	IO_3^-/I^-	λ > 300 nm 450 W Hg lamp	-	2
Pt-TiO_2(anatase): Cr,Ta	Pt-TiO_2(rutile): Cr,Ta	IO_3^-/I^-	λ = 420 nm LED	-	3
Pt-$MgTa_2O_{6-y}N_x$/ TaON	PtO_x-WO_3	IO_3^-/I^-	λ ≥ 420 nm 300 W Xe lamp	3.4	4
Ru-$SrTiO_3$:Rh	RuO_2-TiO_2:Ta,N	IO_3^-/I^-	λ ≥ 420 nm 300 W Xe lamp	-	5
Ru-$SrTiO_3$:Rh	IrO_x-$SrTiO_3$:Rh,Sb	Fe^{3+}/Fe^{2+}	λ ≥ 420 nm 300 W Xe lamp	-	6
Ru-$(CuGa)_{0.8}Zn_{0.4}S_2$	$BiVO_4$	$[Co(terpy)_3]^{3+/2+}$	λ ≥ 420 nm 300 W Xe lamp	3.0	7
Pt-$CuGaS_2$	TiO_2	RGO	Full arc 300 W Xe lamp	1.3	8
Pt-$(CuGa)_{0.5}ZnS_2$	RGO-(CoO_x/$BiVO_4$)	LiCl/CsCl	λ ≥ 420 nm 300 W Xe lamp	0.8	9
SWCNT/fullerodendron/ Ru(III)	$BiVO_4$	$[Co(bpy)_3]^{3+/2+}$	λ ≥ 422 nm 300 W Xe lamp	-	10
Ru-$SrTiO_3$:Rh	Fe−H−Cs−WO_3	VO_2^+/VO^{2+}	λ ≥ 420 nm 300 W Xe lamp	4.0	11
Ru/SrTiO3:Rh	$BiVO_4$	Fe^{3+}/Fe^{2+}	λ ≥ 420 nm 300 W Xe lamp	4.2	12
$Rh_yCr_{2-y}O_3$/ZrO_2/TaON	Au/CoO_x-$BiVO_4$	$[Fe(CN)_6]^{3-}$/ $[Fe(CN)_6]^{4-}$	λ ≥ 420 nm 300 W Xe lamp	5	13
Pt/$Ba_{(0.3)}$–Ta_3N_5	PtO_x-WO_3	IO_3^-/I^-	λ ≥ 420 nm 300 W Xe lamp	0.1	14
Pt-$BaTaO_2N$	PtO_x-WO_3	IO_3^-/I^-	λ ≥ 420 nm 300 W Xe lamp	0.06	15
Pt-$BaTaO_2N$ (RbCl)	Cs-PtO_x-WO_3	IO_3^-/I^-		3.4	16
**3%Pt-$Na_{0.56}WO_{3-x}$**	**0.5%Pt-WO_3**	**IO_3^-/I^-**	**λ ≥ 420 nm 300 W Xe lamp**	**6.0**	**This work**

Below a detailed AQY calculation at 420 nm with an O_2 evolution rate of 0.3 μmol h^{-1} is shown

$$= \frac{4 \times \mathbf{0.3} \times 10^{-6} mol \times 6.023 \times 10^{23} mol^{-1}}{(\mathbf{0.5}) \times 10^{-3}\, W\, cm^{-2} \times 3.14 \times (\mathbf{1\, cm})^2)/(\mathbf{2.95} \times 1.6 \times 10^{-19} J)} \times 100\%$$

$$= \frac{\frac{7.23 \times 10^{17}}{3600}}{(1.57 \times 10^{-3})/(4.72 \times 10^{-19})} \times 100\%$$

$$= \frac{0.2 \times 10^{15}}{0.33 \times 10^{16}} \times 100\%$$

$$= 0.0606 \times 100\% = \mathbf{6.06\%}$$

References

(1) Tegg, L.; Cuskelly, D.; Keast, V. J. The Sodium Tungsten Bronzes as Plasmonic Materials: Fabrication, Calculation and Characterization. *Mater Res Express* **2017**, *4* (6), 065703. https://doi.org/10.1088/2053-1591/aa6c40.

(2) Zhao, W.; Maeda, K.; Zhang, F.; Hisatomi, T.; Domen, K. Effect of Post-Treatments on the Photocatalytic Activity of Sm2Ti2S2O5 for the Hydrogen Evolution Reaction. *Phys. Chem. Chem. Phys.* **2014**, *16* (24), 12051–12056.

(3) Tanigawa, S.; Irie, H. Visible-Light-Sensitive Two-Step Overall Water-Splitting Based on Band Structure Control of Titanium Dioxide. *Appl Catal B* **2016**, *180*, 1–5.

(4) Chen, S.; Qi, Y.; Hisatomi, T.; Ding, Q.; Asai, T.; Li, Z.; Ma, S. S. K.; Zhang, F.; Domen, K.; Li, C. Efficient Visible-Light-Driven Z-Scheme Overall Water Splitting Using a MgTa2O6−xNy /TaON Heterostructure Photocatalyst for H2 Evolution. *Angewandte Chemie International Edition* **2015**, *54* (29), 8498–8501. https://doi.org/10.1002/anie.201502686.

(5) Nakada, A.; Nishioka, S.; Vequizo, J. J. M.; Muraoka, K.; Kanazawa, T.; Yamakata, A.; Nozawa, S.; Kumagai, H.; Adachi, S.; Ishitani, O.; Maeda, K. Solar-Driven Z-Scheme Water Splitting Using Tantalum/Nitrogen Co-Doped Rutile Titania Nanorod as an Oxygen Evolution Photocatalyst. *J Mater Chem A Mater* **2017**, *5* (23), 11710–11719.

(6) Niishiro, R.; Tanaka, S.; Kudo, A. Hydrothermal-Synthesized SrTiO3 Photocatalyst Codoped with Rhodium and Antimony with Visible-Light Response for Sacrificial H2 and O2 Evolution and Application to Overall Water Splitting. *Appl Catal B* **2014**, *150–151*, 187–196.

(7) Kato, T.; Hakari, Y.; Ikeda, S.; Jia, Q.; Iwase, A.; Kudo, A. Utilization of Metal Sulfide Material of (CuGa)1–XZn2xS2 Solid Solution with Visible Light Response in Photocatalytic and Photoelectrochemical Solar Water Splitting Systems. *J Phys Chem Lett* **2015**, *6* (6), 1042–1047.

(8) Iwashina, K.; Iwase, A.; Ng, Y. H.; Amal, R.; Kudo, A. Z-Schematic Water Splitting into H2 and O2 Using Metal Sulfide as a Hydrogen-Evolving Photocatalyst and Reduced Graphene Oxide as a Solid-State Electron Mediator. *J Am Chem Soc* **2015**, *137* (2), 604–607.

(9) Yoshino, S.; Iwase, A.; Ng, Y. H.; Amal, R.; Kudo, A. Z-Schematic Solar Water Splitting Using Fine Particles of H2-Evolving (CuGa)0.5ZnS2 Photocatalyst Prepared

by a Flux Method with Chloride Salts. *ACS Appl Energy Mater* **2020**, *3* (6), 5684–5692.
(10) Izawa, T.; Kalousek, V.; Miyamoto, D.; Murakami, N.; Miyake, H.; Tajima, T.; Kurashige, W.; Negishi, Y.; Ikeue, K.; Ohkubo, T.; Takaguchi, Y. Carbon-Nanotube-Based Photocatalysts for Water Splitting in Cooperation with BiVO4 and [Co(Bpy)3]3+/2+. *Chem Lett* **2019**, *48* (5), 410–413.
(11) Miseki, Y.; Fujiyoshi, S.; Gunji, T.; Sayama, K. Photocatalytic Z-Scheme Water Splitting for Independent H2/O2 Production via a Stepwise Operation Employing a Vanadate Redox Mediator under Visible Light. *The Journal of Physical Chemistry C* **2017**, *121* (18), 9691–9697.
(12) Kato, H.; Sasaki, Y.; Shirakura, N.; Kudo, A. Synthesis of Highly Active Rhodium-Doped SrTiO3 Powders in Z-Scheme Systems for Visible-Light-Driven Photocatalytic Overall Water Splitting. *J Mater Chem A Mater* **2013**, *1* (39), 12327–12333.
(13) Qi, Y.; Zhao, Y.; Gao, Y.; Li, D.; Li, Z.; Zhang, F.; Li, C. Redox-Based Visible-Light-Driven Z-Scheme Overall Water Splitting with Apparent Quantum Efficiency Exceeding 10%. *Joule* **2018**, *2* (11), 2393–2402.
(14) Qi, Y.; Chen, S.; Li, M.; Ding, Q.; Li, Z.; Cui, J.; Dong, B.; Zhang, F.; Li, C. Achievement of Visible-Light-Driven Z-Scheme Overall Water Splitting Using Barium-Modified Ta3N5 as a H2-Evolving Photocatalyst. *Chem Sci* **2017**, *8* (1), 437–443.
(15) Dong, B.; Qi, Y.; Cui, J.; Liu, B.; Xiong, F.; Jiang, X.; Li, Z.; Xiao, Y.; Zhang, F.; Li, C. Synthesis of BaTaO2N Oxynitride from Ba-Rich Oxide Precursor for Construction of Visible-Light-Driven Z-Scheme Overall Water Splitting. *Dalton Transactions* **2017**, *46* (32), 10707–10713.
(16) Wang, Z.; Luo, Y.; Hisatomi, T.; Vequizo, J. J. M.; Suzuki, S.; Chen, S.; Nakabayashi, M.; Lin, L.; Pan, Z.; Kariya, N.; Yamakata, A.; Shibata, N.; Takata, T.; Teshima, K.; Domen, K. Sequential Cocatalyst Decoration on BaTaO2N towards Highly-Active Z-Scheme Water Splitting. *Nat Commun* **2021**, *12* (1), 1005.
